# Transcriptomic analyses of *Aedes aegypti* cultured cells and ex vivo midguts in response to an excess or deficiency of heme: a quest for transcriptionally-regulated heme transporters

**DOI:** 10.1186/s12864-020-06981-5

**Published:** 2020-08-31

**Authors:** Heather Eggleston, Zach N. Adelman

**Affiliations:** 1 Genetics Graduate Program, Texas A&M University, College Station, TX 77843 USA; 2 Department of Entomology, 329A Heep Center, Texas A&M University, 370 Olsen Blvd, College Station, TX 77843 USA

**Keywords:** Heme, *Aedes aegypti*, Transcriptomes, RNAi, Transporter, Mosquito midgut

## Abstract

**Background:**

*Aedes aegypti* is the principle vector of many arboviruses, including dengue virus and Zika virus, which are transmitted when an infected female mosquito takes a blood meal in order to initiate vitellogenesis. During blood digestion, ~ 10 mM heme-iron is ingested into the midgut lumen. While heme acts as both a nutrient and signaling molecule during blood digestion, it can also be highly toxic if left unchaperoned. Both signaling by, and degradation of, heme are intracellular processes, occurring in the nucleus and cytoplasm, respectively. However, the precise mechanism of heme uptake into the midgut epithelium is not currently known.

**Results:**

We used next generation RNA sequencing with the goal to identify genes that code for membrane bound heme import protein(s) responsible for heme uptake into the midgut epithelium. Heme deprivation increased uptake of a heme fluorescent analog in cultured cells, while treatment of midguts with an excess of heme decreased uptake, confirming physiological changes were occurring in these heme-sensitive cells/tissues prior to sequencing. A list of candidate genes was assembled for each of the experimental sample sets, which included Aag2 and A20 cultured cells as well as midgut tissue, based on the results of a differential expression analysis, soft cluster analysis and number of predicted transmembrane domains. Lastly, the functions related to heme transport were examined through RNAi knockdown.

**Conclusions:**

Despite a large number of transmembrane domain containing genes differentially expressed in response to heme, very few were highly differentially expressed in any of the datasets examined. RNAi knockdown of a subset of candidates resulted in subtle changes in heme uptake, but minimal overall disruption to blood digestion/egg production. These results could indicate that heme import in *Ae. aegypti* may be controlled by a redundant system of multiple distinct transport proteins. Alternatively, heme membrane bound transport in *Ae. aegypti* could be regulated post-translationally.

## Background

The *Aedes aegypti* mosquito is an insect of substantial medical importance as it vectors viruses such as dengue, chikungunya, yellow fever and Zika, resulting in human morbidity and mortality [1–4]. Viral transmission is dependent on the female ingesting infected vertebrate blood, upon which the virus must pass multiple infection barriers in order to allow for transmission to the next vertebrate host [5]. Once obtained, the bloodmeal taken by *Ae. aegypti* is digested by enzymes secreted from the midgut epithelium over the course of the 72-h digestion process [6–9]. Two key sources of nutrients obtained from these proteins include amino acids and heme-iron. The amino acids are utilized as building blocks by the mosquito to produce the lipids, carbohydrate and egg proteins necessary for vitellogenesis [10]. While heme acts as both a source of iron that is deposited into the developing eggs [11], it is also a signaling molecule resulting in the activation of vitellogenesis through the activation of the steroid hormone 20-hydroxyecdysone by binding to and stabilizing the nuclear receptor E75 [12–16].

While heme is a vital nutrient, it is also a potent toxin as its pro-oxidant nature leads to the production of reactive oxygen species when left unchaperoned. As a consequence, the female mosquito has developed strategies to limit heme toxicity during blood digestion, beginning with the peritrophic matrix (PM), which is synthesized by the midgut epithelium after blood ingestion and acts as a barrier isolating the epithelial cells from the toxic products of digestion [17]. Comprised of proteins, proteoglycans, and chitin fibrils [18, 19], at least one PM protein has been reported to bind heme, *Ae. aegypti* intestinal mucin 1 (AeIMUCI) [20, 21]. Heme toxicity is further reduced through its degradation by heme oxygenase in the cytosol of the epithelial cells into biliverdin IX α (BV α), iron and CO [for reviews, see [22, 23]]. Evidence of heme transport into the cytosol and that of its degradation product, biglutaminyl-biliverdin, back out into the lumen was observed by Pereira et al., (2007) who found a color change in the lumen over the course of digestion from heme red to bilin pigment green [24]. Although heme is known to enter the midgut epithelium and its degradation product to exit back into the lumen for excretion, the mechanisms responsible for this transport are unknown.

As the primary membrane-bound heme transporters characterized in vertebrates do not appear to be conserved in *Ae. aegypti* [25], de novo identification and characterization of novel transporters is needed. We reasoned that similar to prior work identifying novel heme transporters in *C. elegans* [26], treatment of *Ae. aegypti* cells with an excess of heme, or with media lacking heme could result in diagnostic transcriptional changes as an attempt to both control heme bioavailability and to limit heme toxicity (heme homeostasis). Bottino-Rojas et al. measured transcriptional changes in an *Aedes aegypti* cell line in response to heme [27]. Our study builds upon these findings by incorporating multiple cell lines and time points as well as measuring the transcriptional responses of ex vivo dissected midguts exposed to heme. Transcriptomic analysis of these cell lines and midgut tissue resulted in the identification of differentially expressed transcripts, including those containing multipass transmembrane domains associated with potential transport of heme. However, no single gene was found significantly differentially expressed in all conditions, suggesting redundancy in heme hemostasis. A subset of the genes found differentially expressed in both midgut epithelial cells and cultured cells were analyzed through RNAi knockdown; while minor changes in heme uptake were observed, no single gene was found responsible for uptake ability.

## Results

### ZnMP visualization of heme import changes in response to heme overload or deficiency

**Fig. 1:**
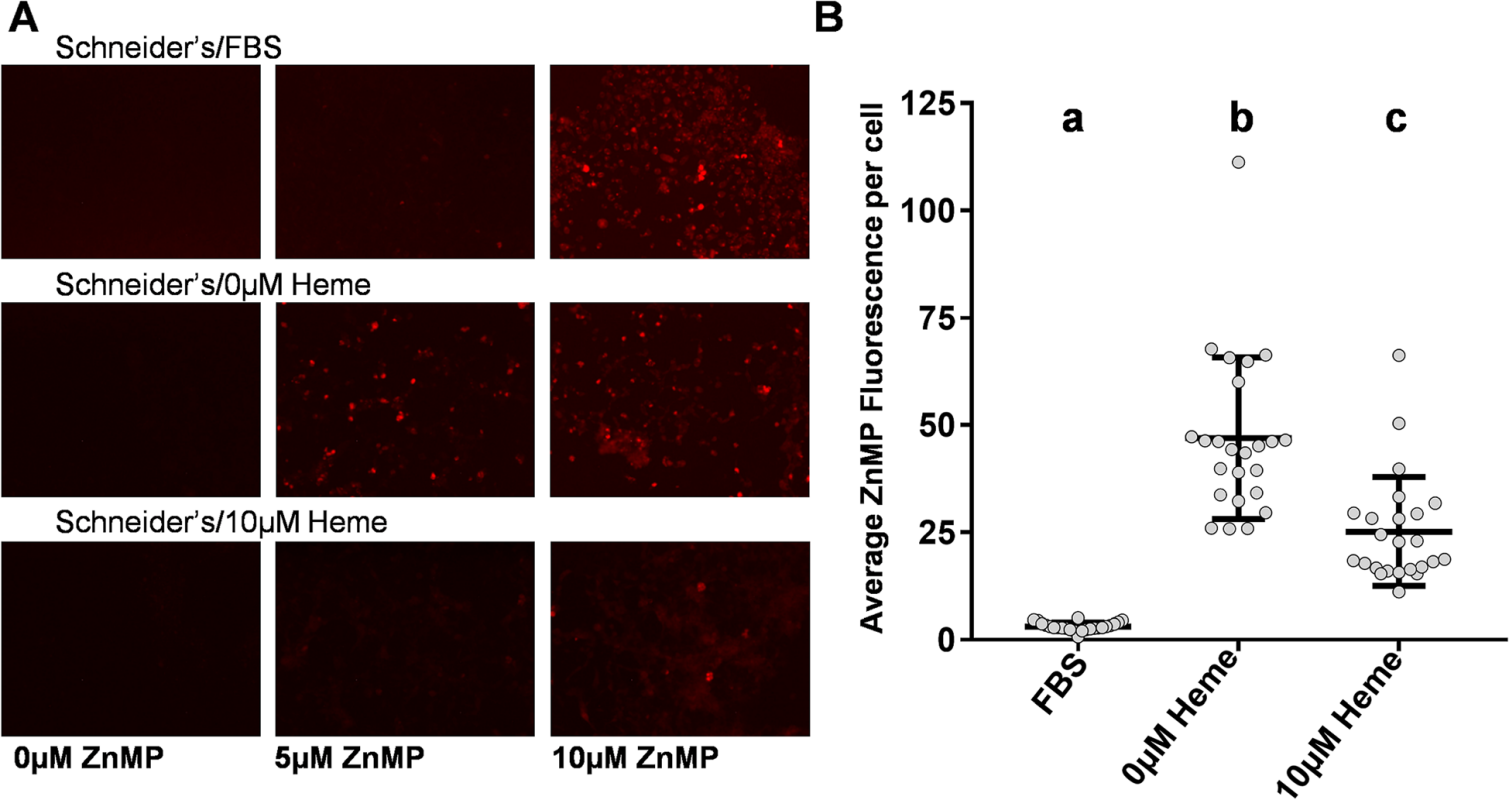
Heme deficiency in Aag2 cells increases ZnMP uptake. **a** Images of ZnMP fluorescence in cells incubated in various heme concentrations. Brightness and contrast were increased uniformly in all images by reducing the pixel range from 0 to 255 to 0–150. **b** Quantitative measurement of 5 μM ZnMP normalized fluorescence of cells exposed to heme treatments. Each dot represents the average normalized ZnMP fluorescence of a well of a 96-well plate after 30-min incubation. The middle horizontal line on each treatment group represents the mean and the lines above and below represent ±1 SD. Each heme treatment was distinct from each other (q-val < 0.01) and thus labeled a, b &c

As no heme transport proteins have been identified in any dipteran, we sought to develop an assay to rapidly assess heme transport ability in mosquito cells. In vertebrates, transport of Zinc Mesoporphyrin (ZnMP), a heme fluorescent analog, occurs through the same active transport system as heme [28], indicating that monitoring ZnMP fluorescence could be used to examine changes in mosquito heme transport systems. In mammalian cells, ZnMP could not be degraded by heme oxygenase [29], which allowed for the accumulation of its fluorescent signal over time [28]. In this study, we examined ZnMP fluorescence in Aag2 cells after treatment over a 72-h period with regular growth media (10% FBS, no added heme), heme-deficient media (no FBS, no added heme) or heme-supplemented media (no FBS, 10 μM heme). We observed ZnMP fluorescence following incubation in 10 μM ZnMP regardless of the heme-treatment, indicating Aag2 cells are capable of uptake (Fig. 1a). However, only the heme-deficient treatment had detectable fluorescence when incubated in 5 μM ZnMP, indicating an increased ZnMP uptake capacity under heme-deficient conditions. Quantitative analysis of these cells confirmed that heme-starved cells showed increased cellular uptake when compared to either the heme-supplemented cells or those grown in regular media (Fig. 1b). We also note that the heme-supplemented cells showed higher ZnMP uptake than those in regular growth media, suggesting that heme present in the regular growth media exceeds 10 μM due to the addition of FBS.

### RNA sequencing and differential expression analysis of cultured cell samples exposed to heme-deficiency and heme-overexposure conditions

Having observed changes in heme uptake through ZnMP fluorescence in these conditions, we isolated total RNA from heme-treated cells, extracted mRNA and performed RNA-seq with the goal to observe heme-induced transcriptomic changes. In order to identify differentially expressed genes across cell types and treatment regimens, three sets of experimental conditions were examined in Aag2 cells and one in A20 cells (Additional file 4, Fig. S1; Table 1). We describe below the results of the transcriptomic analyses performed on Aag2 cells grown in Schneider’s *Drosophila* media.

**Table 1:** *Ae. aegypti* cultured cell heme exposure experimental conditions and differential expression/cluster analysis results

Cell Type	Incubation Time	Type of Growth Media for Experimental Incubation Time			Differential Expression Comparisons [genes (genes w/TM domains)]				Cluster Analysis [genes (genes w/TM domains)]	
		Normal	Heme-Deficient	Heme-Overexposure	Normal vs Normal + 20 μM Heme	Normal vs 0 μM Heme	Normal vs 10 μM heme	0 μM vs 10 μM heme	Import-like clusters	Export-like clusters
Aag2	48 h	Schneider’s *Drosophila* + FBS	N/A	Schneider’s *Drosophila* + FBS + 20 μM Heme	551 (211)				1611(455)	1391(385)
Aag2	72 h	Schneider’s *Drosophila* + FBS	Schneider’s *Drosophila*	Schneider’s *Drosophila* + 10 μM Heme		587 (251)	1990 (737)	2274 (856)	745(238)	687(240)
Aag2	72 h	Leibovitz’s L-15 + FBS	Leibovitz’s L-15	Leibovitz’s L-15 + 10 μM Heme		4166 (1218)	4979 (1568)	3200 (1121)	905(229)	670(197)
A20	72 h	Leibovitz’s L-15 + FBS	SF900 II	SF900 II + 10 μM Heme		745 (300)	2815 (658)	3692 (942)	1109(448)	757(143)

**Fig. 2:**
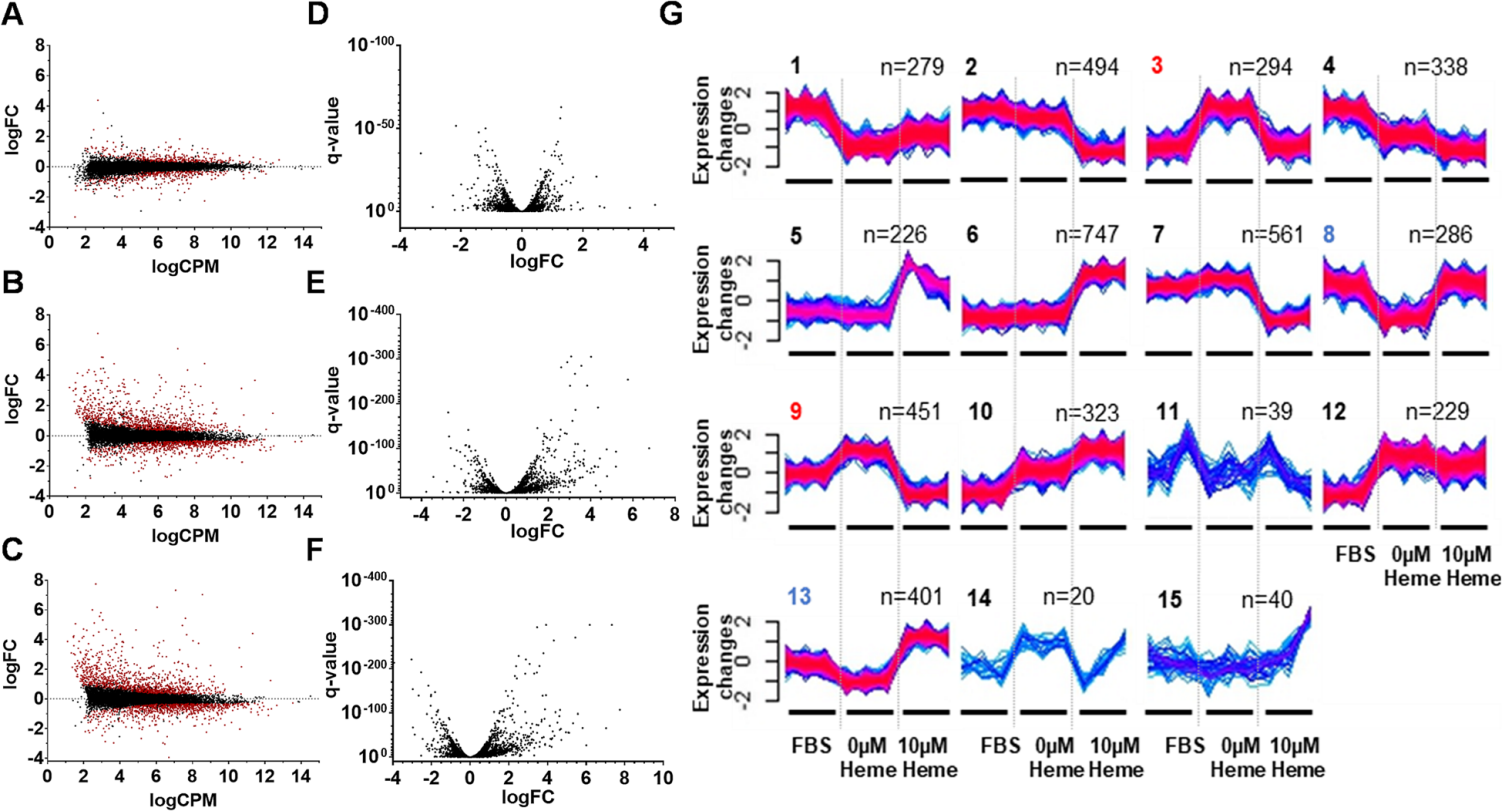
RNAseq-based transcriptomic analyses after 72-h heme treatment in Aag2 cells in Schneider’s *Drosophila* medium. Aag2 cells grown with FBS, without FBS (0 μM heme) or without FBS+ 10 μM heme (**a-c**) Log2 fold change (LogFC) vs Log10 counts per million (LogCPM) graph of genes found differentially expressed in analysis for each comparison. Genes with an adjusted *p*-value corrected for multiple testing (False discovery rate: FDR) of < 0.0001 are shown in red. **d-f** Volcano plot of genes found differentially expressed. **a**&**d** FBS vs 0 μM heme (**b**&**e**) FBS vs 10 μM Heme (**c&f**) 0 μM heme vs 10 μM heme. **g** 15 gene expression profile clusters were identified through soft clustering. Only genes with membership scores > 50% are shown in each cluster. Red lines indicate high membership of a gene in a particular cluster, while blue indicates low membership. Heme import-like clusters are highlighted in red and export-like clusters in blue. The different heme treatment groups are given on the X-axis of each cluster. n, the number of genes present in a cluster

In heme-exposed Aag2 cells grown in Schneider’s *Drosophila* medium, 8644 annotated transcripts were considered expressed across all samples, with biological replicates clustering together and treatment groups (Regular growth media, 0 μM heme & 10 μM heme) distinctly separate (Additional file 4, Fig. S2). Of these, 587 genes were found significantly differentially expressed in the regular growth media vs 0 μM heme comparison [Table S1 (Additional file 1); q-value < 0.0001; Fig. 2a, d]. For the other comparisons, 1990 genes were found differentially expressed in the normal media vs 10 μM heme comparison (Fig. 2b, e) and 2274 when comparing the 0 μM heme and 10 μM heme groups (Fig. 2c, f). As we were most interested in membrane-bound transporters, transmembrane (TM) domain predictions were performed on all DE genes; 251, 737 and 856 were identified respectively with at least 1 TM domain. Interestingly, for all comparisons the number of differentially expressed genes with predicted TM domains was significantly more than expected by chance alone (obs 251, exp. 185; obs 737, exp. 638; obs 856, exp. 729; all *p*-values < 0.00001). Despite the large number of DE genes, we note that the magnitude of transcriptional change of TM-containing genes was overall quite small. When genes that contained 2 or more TM domains and a greater than 2-fold change between heme treatment types were examined, only 2 were downregulated in response to heme deficiency and upregulated in response to heme exposure: AAEL009206 and AAEL009813. AAEL009206 is described as an organic cation transporter, while AAEL009813 is annotated as glutamate receptor 7.

Similar analyses were performed on the other sets of experimental conditions (48-h heme-treated Aag2 cells, 72-h heme-treated A20 cells and 72-h heme-treated Aag2 cells in L-15; described in Table 1) and are discussed in more detail in the supplemental text (Additional file 3). All of the varying heme treatments in each set of experimental conditions showed similar expression between biological replicates and dissimilar between the treatments types (Additional file 4, Fig. S3A, S4 & S5). The number of DEGs and the amount that contain at least 1 transmembrane domain are given in Table 1 (48-h heme-treated Aag2: Additional file 4, Fig. S3B&C, Table S2; A20: Fig. S6A-F, Table S3; heme-treated Aag2 in L-15: Fig. S7A-F, Table S4; All supplement tables: Additional file 1). In addition, a small number of highly expressed (2-fold up/down) multi-transmembrane domain containing genes with expression patterns consistent with that expected for genes associated with heme transport were found in each dataset; 28 genes in 48-h heme treated Aag2 cells, 98 in 72-h heme treated A20 cells and 13 in 72-h heme treated Aag2 cells in L-15 media. Surprisingly, when we compared the lists of DEG to each other we found no overlap, and so decided to pursue an alternative method of transcriptomic analysis based on cluster analysis.

### Soft cluster analysis of cultured cells exposed to heme-deficiency and heme-overexposure conditions

As an alternative to strict differential expression analysis, we examined expression patterns across each treatment to identify those genes with a pattern of expression consistent with our expectations for transcriptionally-regulated heme transport proteins (Import/Export: upregulated/downregulated upon heme deficiency, downregulated/upregulated upon heme excess). When we examined the transcriptome of 72-h heme-treated Aag2 cells grown in Schneider’s *Drosophila* media, 4728 genes were assigned across 15 distinct cluster patterns (Each gene displayed > 50% membership confidence to its assigned cluster), as shown in Fig. 2g (Table S1). Genes that showed the expected expression pattern for those with heme import function (↑ heme-deficiency, ↓ heme-overexposure) are shown in clusters 3 and 9; these clusters contained 238 genes with 1 or more predicted TM domain (85 and 153, respectively). Clusters 8 and 13 contained genes with the expression pattern consistent with potential heme export function (↓ heme-deficiency, ↑ heme-overexposure) and included 240 genes (99 and 142 respectively) with at least 1 TM domain prediction. Our analysis also appeared to identify clusters associated with gene expression changes due to the alterations in nutrient content in the growth media when fetal bovine serum is present/absent (clusters 1, 4, 10 and 12). Similar cluster-based analyses were performed for other experimental conditions (Table 1) are described in detail in the supplemental text (Additional file 3). For each, the number of genes assigned to the import-like clusters and export-like clusters and the number that contain at least 1 predicted transmembrane domain are given in Table 1 (48-h heme-treated Aag2: Fig. S3D; 72-h heme-treated A20: Fig. S6G; 72-h heme-treated Aag2 in L-15: Fig. S7G).

When the differential expression and the soft cluster analyses were compared to each other for each set of cultured cell conditions (Table 1), many of the heme transporter candidate genes identified in one were also found in the other (Additional file 4, Fig. S8–11). In addition, when the genes found highly differentially regulated (> 2-fold/down; ≥2TM) in each dataset were compared to those identified in the import-like/export-like clusters, the majority were present in both (48-h heme-treated Aag2: 28/28; 72-h heme-treated A20: 84/98; 72-h heme-treated Aag2 in L-15: 12/13). This commonality between the two different analyses indicated that these approaches converged on a single set of differentially regulated genes. In the end, as the majority of the highly expressed multipass TM containing DEGs were also present in import-like or export-like clusters for each dataset, we considered that these genes might play a role in the cellular response to heme. For example, both of the genes found highly differentially expressed (AAEL009206 & AAEL009813) were also present in the export-like clusters.

We reasoned that genes undergoing similar expression changes upon heme treatment/deficiency across different media types (L-15 or Schneider’s) or cell types (Aag2 or A20) would be the strongest candidates for a role in heme homeostasis. We identified those genes shared between experiments (Table 1) based on their expression pattern (cluster analysis) and number of predicted TM domains (≥1 TM domains) (Additional file 4, Fig. S12) and found that the majority of genes in expression clusters consistent with heme export (*n* = 649) or import (*n* = 767) function were unique to only 1 of the 4 datasets (Table 2). However, considering import-like gene expression patterns, 223 genes were identified in 2/4 datasets, 46 genes were identified in 3/4 datasets and just two (AAEL006432 and AAEL014003) were identified by cluster analysis in all 4 cell culture experiments. Likewise, a small number of genes displayed heme export-like expression patterns in multiple datasets, with 136 genes found in 2/4 datasets and 11 in 3/4 datasets. No genes with the expression pattern expected for a heme export function were found in all 4 datasets.

**Table 2:** Genes Present in Import-like or Export-like Clusters Across Multiple *Ae. aegypti* Cultured Cell Datasets

	Genes Present in One or More Datasets			
	only 1 dataset	2 datasets	3 datasets	4 datasets
Genes present in clusters consistent with heme export	649	136	11	0
Genes present in clusters consistent with heme import	767	223	46	2

### Comparing candidate genes to those identified in previously published analyses

As we observed substantial variability in the suite of differentially regulated genes between cell line type and even growth media conditions in response to heme, we sought to compare our candidate lists with other published experiments. DEGs and the genes present in the import-like/export-like clusters were compared to previous analyses performed in Aag2 cells [27] and bloodfed *Ae. aegypti* females [30] to identify genes shared between them. Bottino-Rojas [27] found 28 DEG (≥2-fold/down, ≥2TM) in Aag2 cells exposed to heme for 24 h that contained predicted functions related to transport. Of these genes, 22 were also identified as a DEG and/or present in an import-like/export-like cluster in at least one of our cultured cell experiments (7 genes in all 4 datasets, 3 genes in 3 datasets, 8 genes in 2 datasets & 4 genes in just 1 dataset). The functions of some of these 22 shared genes include 3 ABC transporters, 1 member of the ZIP family of zinc transporters (AAEL014762) and 1 cystinosin (AAEL006113) and 7 of unknown function. AAEL014762 is an iron transporter localized in the endocytic vesicles and is important in exporting iron from the midgut [31, 32]. Cystinosin is involved in the transport of cystine into the cytosol which then serves as a site for redox reactions to occur, indicating its important role in heme-generated ROS mitigation. Thus, the remaining genes present across multiple analyses may represent promising candidates as heme transporters. Likewise, of the ~ 369 multipass TM containing genes found highly differentially expressed in bloodfed vs sugar fed females (> 1 log2 or < − 1 log2; ≥100 reads in at least 1 sample) [30], almost half (171, 46%) were also identified as a DEG and/or present in an import-like/export-like expression cluster in one or more of our cultured cell experiments. In fact, the gene AAEL009853 (trysin/serine protease) was differentially expressed in both A20 cells in the absence of the FBS (0 μM heme samples) and bloodfed females and is involved in the digestion of proteins. In addition, the cytinosin AAEL006113 was also upregulated in both bloodfed midguts and heme-exposed cells (A20 &Aag2) indicating the possibility of a shared ROS response pathways between them. Altogether, these analyses highlight both the plasticity of the *Ae. aegypti* transcriptional response to heme deficiency/excess and a number of candidate genes that might serve as heme transporters. As no single candidate gene appeared dominant across all experiments, we sought further evidence of transcriptional response to heme directly in the mosquito midgut prior to beginning reverse genetic analyses of candidate genes,

### ZnMP uptake in *Aedes aegypti* midguts

**Fig. 3:**
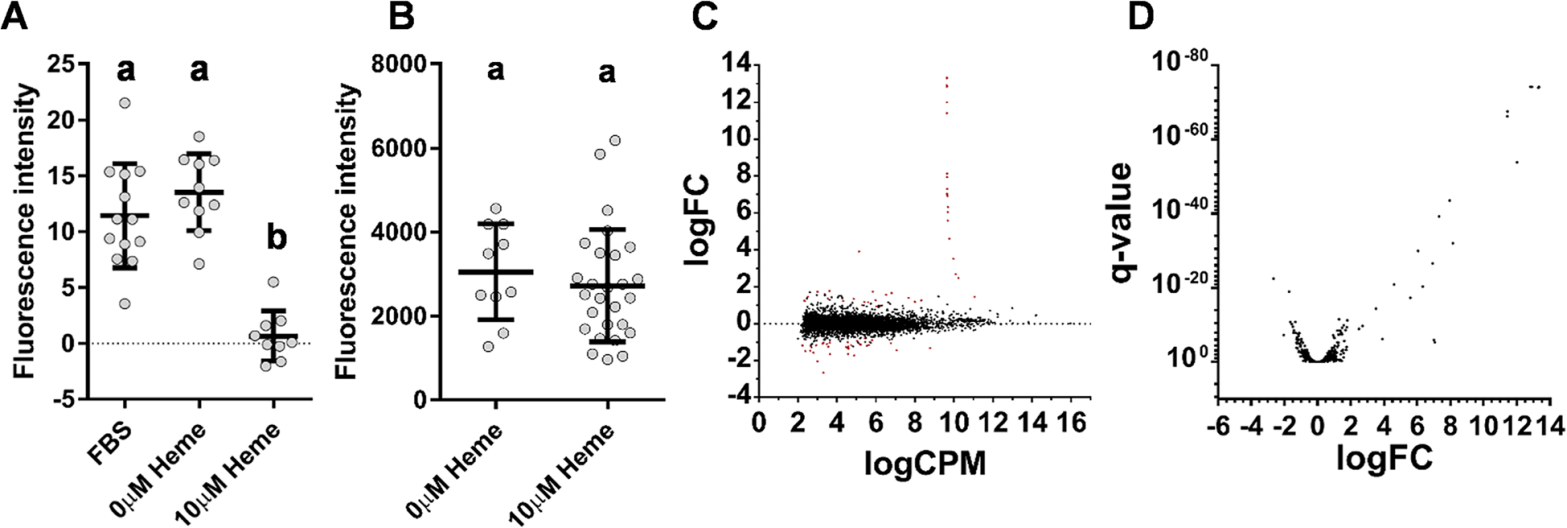
Heme treatment reduces ZnMP uptake and alters gene expression in *Aedes aegypti* female midguts. *Ae. aegypti* female midguts were incubated in 0 μM or 10 μM hemin chloride for 35 h. **a** Background corrected ZnMP fluorescence measurements of each dissected midgut. The middle horizontal line on each treatment group represents the mean and the lines above and below represent ±1 SD. Statistical significance shown by the labels a&b. **b**. Background corrected pHrodo^TM^ Green Dextran fluorescence of dissected midguts. Heme deficient and exposed midgut fluorescence not significantly distinct (*p*-value = 0.6316) **c** Log2 fold change (LogFC) vs Log10 counts per million (LogCPM) graph of genes found differentially expressed. Genes with an adjusted *p*-value corrected for multiple testing of < 0.00001 (false discovery rate: FDR) are shown in red. **d** Volcano plot of genes found differentially expressed in analysis

As *Ae. aegypti* midgut epithelial cells are directly involved in heme uptake and digestion following blood feeding [33], we decided to examine their transcriptional responses upon heme exposure. As a typical blood meal taken in by the female mosquito is composed of many different components present in vertebrate blood, we attempted to disentangle their effects on the midgut by using a protein-free artificial bloodmeal [with either 0 mM or 10 mM heme; protocol from [34]]. We initially chose 10 mM heme concentration as that would be the maximum amount of heme present in a drop of blood that could be released at once [18], however smaller concentrations as low as 625 μM were also examined. Nevertheless, this method was not effective due to the failure to solubilize heme in aqueous feeding solutions and the corresponding toxicity of solvent such as DMSO. Therefore, an ex vivo application was performed with midguts incubated post-dissection in either regular growth cultured cell media, heme-deficient media (0 μM heme) or heme-overload media (10 μM heme) followed by a ZnMP fluorescence assay based on the adapted insect cultured cell assay [28]. Following ZnMP incubation, the heme-treated midguts showed substantially decreased uptake of the fluorescent analog compared to those incubated in regular or heme-deficient media (q-value< 0.0001; Fig. 3a, S13). However, unlike our observations in cultured cells, no significant difference was observed in ZnMP fluorescence between the normal growth media and heme-starved midguts (q-value = 0.3903). The decreased ZnMP uptake we observed in the 10 μM heme treatment indicated that this concentration was sufficient to alter the physiological readiness of the midguts in a manner similar to what might occur naturally during blood digestion. We considered the possibility that this reduction in ZnMP fluorescence was due to a more general decrease in active transport, rather than a specific downregulation of heme transport. To test this possibility, heme-exposed midguts were incubated with pHrodo™ green dextran as a marker for active endocytosis (Fig. 3b). No difference in fluorescence was observed between the heme deficient/overexposure midguts, suggesting that endocytosis is active upon heme-exposure and that the heme transport pathway(s) is specifically affected.

### RNA sequencing and differential expression analysis of Heme-treated Midguts

As the ability of the midgut to uptake the heme analog ZnMP was reduced after incubation with heme, we hypothesized that this was due to modulation of the gut transcriptome, as the cells temporarily downregulated genes involved in heme import and/or upregulated genes involved in heme export similar to what was observed in *C. elegans* [26]. To test this hypothesis, we performed a differential expression analysis. Few differentially expressed genes were identified when using the strict q-value (false discovery rate) as in the cultured cell datasets (65 DEG, q-value = 0.0001). However, as substantially more variation was observed between the biological replicates in this experiment (Additional file 4, Fig. S14) we considered less stringent q-values as well (Table 3; Additional file 1, Table S5). We found as many as 243 (q-value = 0.05) significant DE genes with 125 downregulated and 118 upregulated genes in response to heme exposure (Fig. 3c&d). Of these, 44% (108) contained at least one predicted TM domain, with 60 downregulated and 48 upregulated (Additional file 1, Table S5). As observed previously in cultured cells, genes encoding at least one TM domain were much more likely to be differentially regulated in response to heme exposure than expected by chance alone (obs 108, exp. 80; *p*-value< 0.00001). Unexpectedly, we identified that 11 of the most highly upregulated genes were mitochondrial in origin (logFC range 2.7–12) and were highly expressed across all the samples (logCPM ~ 10). These mitochondrial genes were not considered further, as we sought to focus on genes encoding factors that might shuttle diet-derived heme through the cellular membranes. This left 58 DEGs with 2 or more TM domains, 34 of which were downregulated and 24 upregulated in the presence of heme. This list was further reduced by removing from consideration those gene products with functions not related to transport, such as those with enzymatic activity or those related to the ROS stress response (Genes remaining: 40; 28 downregulated and 12 upregulated; Table 4). Of the 40 DEG candidates, only 11 were highly differentially regulated when exposed to heme overload conditions, 2-fold decrease (6) or increase (5).

**Table 3:** **The number of significant genes found at different** ***α*** **values**

Adjusted ***α***	# of genes	# of TM genes
0.0001	65	38
0.001	84	47
0.01	154	76
0.05	243	108

**Table 4:** Multipass TM domain containing genes with expression patterns consistent with genes encoding heme transport proteins

NAME	log2FC	q-value	# TM
AAEL000859 protein_coding	−1.3184	1.40E-08	2
AAEL019869 protein_coding	−1.2359	1.05E-05	2
AAEL002557 cationic amino acid transporter	−1.1718	9.42E-07	12
AAEL024460 protein_coding	−1.1243	0.00115	4
AAEL020936 protein_coding	−1.0454	6.14E-05	2
AAEL021618 protein_coding	−1.032	0.02785	2
AAEL024267 protein_coding	−0.982	0.00343	2
AAEL003619 sodium/chloride dependent amino acid transporter	−0.9543	0.00042	12
AAEL001938 ATP-binding cassette sub-family A member 3	−0.8521	0.00624	17
AAEL007191 amino acid transporter	−0.8341	0.00233	10
AAEL010905 protein_coding	−0.826	0.00082	6
AAEL005013 protein_coding	−0.789	0.00148	2
AAEL012440 sodium-bile acid cotransporter	−0.7835	4.60E-05	10
AAEL001495 protein_coding	−0.7597	0.01199	6
AAEL004513 neurotransmitter gated ion channel	−0.7512	0.00589	3
AAEL008664 protein_coding	−0.7068	0.00188	2
AAEL006480 protein_coding	−0.7036	0.00573	3
AAEL014355 symbol, putative	−0.6857	0.04023	2
AAEL003618 sodium/chloride dependent amino acid transporter	−0.6854	0.04341	12
AAEL009531 niemann-pick C1	−0.6551	0.00052	14
AAEL012226 protein_coding	−0.6385	0.02752	4
AAEL009112 protein_coding	−0.6322	0.03157	2
AAEL011918 protein_coding	−0.6194	0.02778	2
AAEL027190 protein_coding	−0.5945	0.00269	8
AAEL000461 protein_coding	−0.5927	0.00551	3
AAEL000417 monocarboxylate transporter	−0.5895	0.02177	12
AAEL003318 oligopeptide transporter	−0.5479	0.01182	10
AAEL021771 protein_coding	−0.5258	0.03568	3
AAEL006855 UDP-galactose transporter	0.469	0.04311	10
AAEL012395 ATP-binding cassette transporter	0.5554	0.02778	12
AAEL023104 protein_coding	0.6348	0.02177	4
AAEL027424 protein_coding	0.7912	0.00758	8
AAEL018150 protein_coding	0.8105	0.03276	13
AAEL022465 protein_coding	0.8262	0.02191	3
AAEL028119 protein_coding	0.8301	0.01236	2
AAEL002129 protein_coding	1.0183	3.69E-05	2
AAEL005043 ATP-dependent bile acid permease	1.1916	8.42E-05	17
AAEL002599 protein_coding	1.2418	3.07E-05	2
AAEL021284 protein_coding	1.6055	1.95E-06	2
AAEL029008 protein_coding	1.7825	5.04E-12	2

**Fig. 4:**
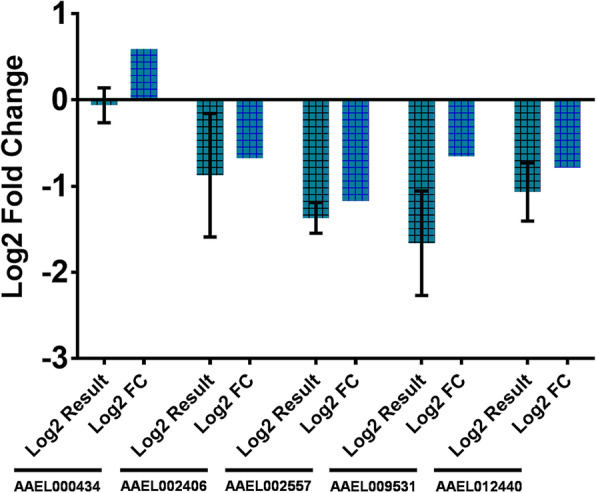
RT-qPCR of a subset of potential heme transport genes in Aag2 cells in response to heme exposure or deficiency. Real time quantitative PCR of 5 genes differentially expressed in midguts treated with 10 μM heme when compared to 0 μM heme control were selected to validate computational results; AAEL000434, AAEL002406, AAEL002557, AAEL009531 and AAEL012440. Teal bars indicate RT-qPCR relative quantification values and blue bars indicate computational fold change values

In order to confirm that the differentially expressed genes identified in this analysis were in fact transcriptionally regulated in heme exposed *Ae. aegypti* midguts, we performed quantitative real-time PCR (RT-qPCR) on a small subset of these genes. The computationally derived fold change values of 5 TM-containing genes with increased/decreased expressionin the presence of heme were compared to the relative quantification (RQ) RT-qPCR values (Fig. 4). In 4 out of the 5 genes examined AAEL002406, AAEL002557, AAEL009531 and AAEL012440 the RT-qPCR results were consistent, all of which showed downregulation in response to heme exposure. Of the 5 genes examined, 2 contained q-values above the α considered in the differential expression analysis (0.05), while one of these, AAEL002406 (0.070), showed consistant downregulation in response to heme exposure, the RT-qPCR results of the other, AAEL000434 (0.064), were not consistant with the upregulation observed previously. These results indicate that the true false discovery rate of this experiment is above the α chosen for this analysis.

### Comparison of Midgut Heme transporter candidates to those found in cultured cells

We compared the list of genes differentially expressed in the midgut upon heme treatment to those genes identified as differentially expressed in the cultured cell analyses with the goal to identify any common heme response mechanisms shared between the distinct cell types examined. In total, 15 of the 40 DEG identified in the midgut were also present in one or more cultured cell datasets. When examining those 28 genes downregulated in the presence of heme (expression pattern consistent with heme import function), 11 were also identified in at least one cell culture experiment [8 were identified in only 1 cell dataset, 1 in 2 datasets and 2 in 3 datasets (Table 5)]. Likewise, 4 of the 12 midgut genes upregulated in the presence of heme (expression consistent with heme export function) were also identified in one cultured cell dataset (Table 5).

**Table 5:** Midgut heme transporter candidates found also in one or more cultured cell dataset

Potential importers				Potential exporters			
Midgut + 1	+ 2	+ 3	+ 4	Midgut + 1	+ 2	+ 3	+ 4
AAEL012226	AAEL011918	AAEL012440		AAEL027424			
AAEL021771		AAEL001495		AAEL028119			
AAEL019869				AAEL006855			
AAEL005013				AAEL012395			
AAEL006480							
AAEL003619							
AAEL000461							
AAEL000417							

### RNAi of *Aedes aegypti* cultured cells

**Fig. 5:**
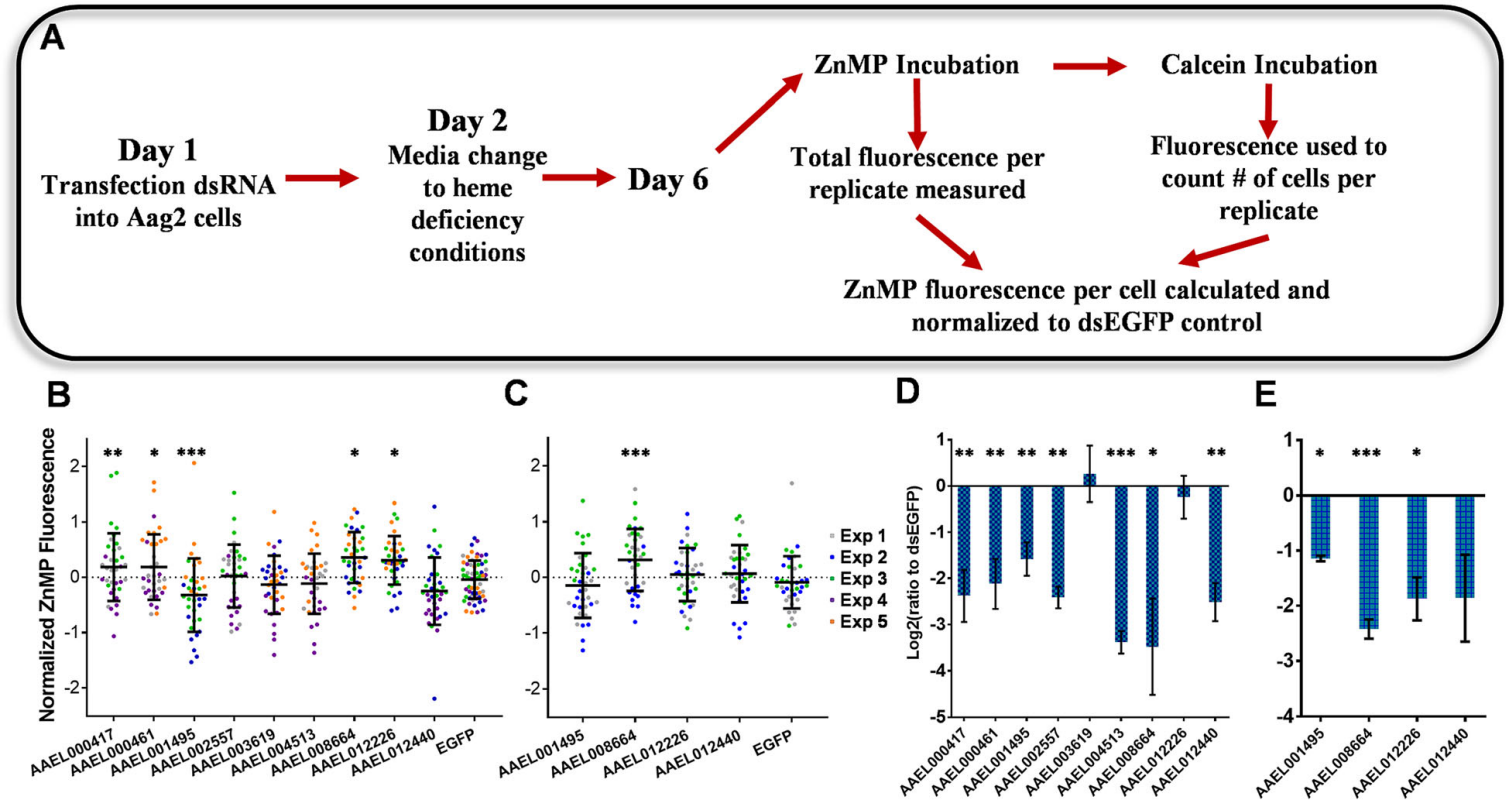
dsRNA knockdown of candidate heme importers in *Aedes aegypti* cultured cells. **a** RNAi knockdown of heme transporter candidate genes in Aag2 cells. **b** Gene targets: AAEL000417, AAEL000461, AAEL001495, AAEL002557, AAEL003619, AAEL004513, AAEL008664, AAEL012226, & AAEL012440. Each point on the graph was the average ZnMP fluorescence per cell normalized to the EGFP control for a knockdown replicate. The colors of each point represent the specific experimental day the point was measured. **c** RNAi with new dsRNA targeting new regions of genes found to effect ZnMP uptake in (**b**). **d** RT-qPCR confirmation of RNAi knockdown of DE genes found in cultured cells treated with heme overload or deficiency. **e** RT-qPCR confirmation of genes knocked down in (**d**). * Indicates genes that exhibited significantly different fluorescence/RQ than control with *p*-values below 0.05, ** *p*-values < 0.01 and *** *p*-values < 0.001

We next sought to determine whether any of differentially regulated genes has a functional role in heme transport (Table 6). We selected 9 candidate DEG found in both the heme-exposed midgut epithelial cells and at least one cultured cell experiment and performed an RNAi knockdown screen in Aag2 cells, measuring the normalized ZnMP fluorescence in the presence/absence of each gene product (Fig. 5a). RT-qPCR of each gene confirmed successful knockdown for 7/9 candidate genes (Fig. 5d). Knockdown of the gene AAEL001495 (*p*-value = 0.0004) resulted in a reduction of ZnMP fluorescence (19.9%) indicating a potential role in heme import (Fig. 5b). Conversely, knockdown of the genes AAEL012226 (*p*-value = 0.0363), AAEL008664 (*p*-value = 0.0104), AAEL000417 (*p*-value = 0.0036) and AAEL000461 (*p*-value = 0.0356) resulted in a modest increase in ZnMP fluorescence when compared to EGFP (23.8, 28.4, 13.8 and 13.8% respectively) indicating their possible role in heme export. As knockdown of AAEL003619 and AAEL012226 were not confirmed, their potential role in heme transport remains unresolved.

**Table 6:** Cultured cell candidates selected for RNAi knockdown in Aag2 cells

Gene	Annotation	# TM	Dataset Identified in	Type of transport
AAEL000417	monocarboxylate transporter	12	A20	Import
AAEL000461	protein_coding	3	A20	Import
AAEL001495	protein_coding	6	Aag2 48 h/A20/Aag2Schn	Import
AAEL002557	cationic amino acid transporter	12	Aag2 48 h	Export
AAEL003619	Na/Cl dependent amino acid transporter	12	Aag2 L15	Import
AAEL004513	neurotransmitter gated ion channel	3	Aag2 48 h	Export
AAEL008664	protein_coding	2	Aag2 48 h/Aag2Schn	Export
AAEL012226	protein_coding	4	Aag2 48 h/Aag2Schn	Import/Export
AAEL012440	sodium-bile acid cotransporter	10	Aag2 48 h/A20/Aag2L15	Import

As we observed significant changes in ZnMP fluorescence for 5 candidate genes when knocked down in Aag2 cells, we designed new dsRNAs targeting independent regions of the top 3 genes in order to confirm their role in heme transport. In this RNAi screen the genes AAEL001495 (decreased fluorescence previously), AAEL008664 (increased) and AAEL012226 (increased) as well as AAEL012440 which showed a non-significant downward trend higher in magnitude than some significant genes (15.8% decrease; *p*-value = 0.2813) were knocked down (Fig. 5b). RT-qPCR of each gene confirmed successful knockdown in all but AAEL012440 (Fig. 5e). The results of this analysis, shown in Fig. 5c, confirmed only AAEL008664 as having a potential role in heme transport, specifically heme export, as a modest increase in ZnMP fluorescence (24.4%; *p*-value = 0.00059) was observed in both screens.

### RNAi of *Aedes aegypti* Midguts

**Fig. 6:**
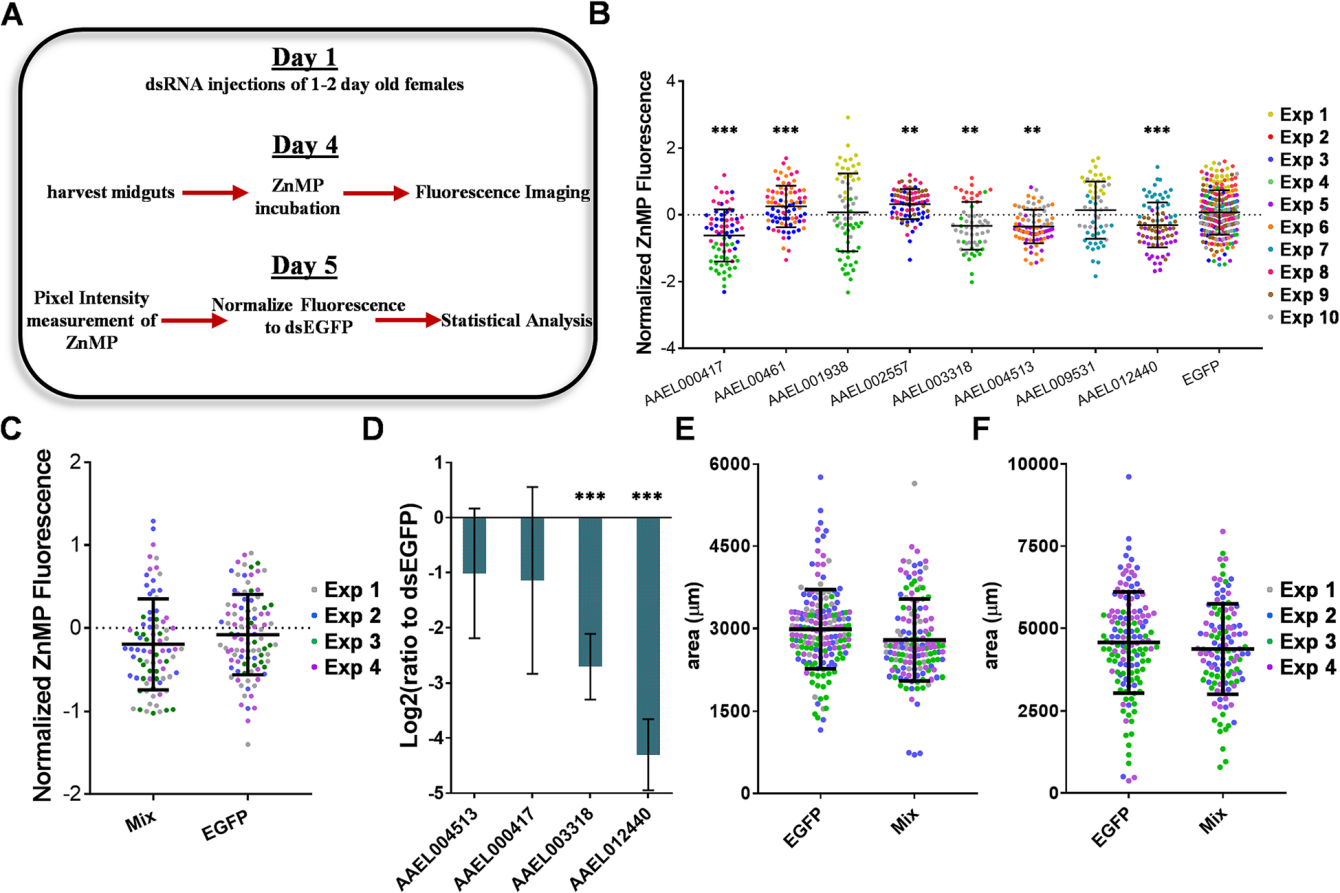
dsRNA knockdown in adult *Aedes aegypti* female midguts of candidate genes. **a** RNAi knockdown of the *Ae. aegypti* genes AAEL000417, AAEL000461, AAEL001938, AAEL002557, AAEL003318, AAEL004513, AAEL009531 and AAEL012440 in *Ae. aegypti* female midguts. **b** ZnMP fluorescence observed after RNAi knockdown of candidate heme transporter genes. Each colored point represents the average ZnMP fluorescence across a midgut normalized to an EGFP control. The colors of each point represent the specific experimental day that the point was observed; each experiment consisted of a sample size of ~*n* = 30 for each gene. ** Indicates genes that exhibited significantly different fluorescence than control with *p*-values below 0.01 and *** with *p*-values < 0.001. **c** ZnMP fluorescence of simultaneous RNAi knockdown of 4 genes, AAEL000417, AAEL003318, AAEL004513 and AAEL012440 in adult female mosquitoes. **d** AAEL003318 & AAEL012440 showed RT-qPCR confirmation of RNAi knockdown as they were distinct from the expression in cells targeted with dsEGFP (*** = p-val < 0.001). (**e**&**f**) Reproductive potential was measured through area measurements of ovaries at (**e**) 12 h and (**f**) 24 h post blood feeding. A slight decrease was observed at 12 h in the mix group (p-val = 0.0191) while no difference was observed at 24 h (p-val = 0.2723)

As no clear single candidate gene emerged from our cell culture-based RNAi screen, we repeated the screen using ex vivo dissected midguts under the assumption that redundancy in heme transport might be reduced in this tissue as compared with undifferentiated cells. To do this, we injected dsRNA targeting one of eight DEG with expression patterns consistent with involvement in heme transport, dissected the midguts and measured ZnMP uptake (Table 7, Fig. 6a&b). Of the 8 genes selected, 5 were found differentially expressed in both midgut epithelial cells and at least one cultured cell experiment, while 3 were DE unique to the midgut epithelial cells. The RNAi-based knockdown of 4 genes resulted in a decreased in ZnMP fluorescence (*p*-value≤0.001) with the highest reduction occurring during the knockdown of AAEL00417 (34.9%), indicating a potential role in heme import. Correspondingly, 2 genes showed an increase in ZnMP fluorescence upon knockdown (*p*-value≤0.0049) with the highest increase occurring in AAEL002557 (25.2%) and thus may play a role in heme export.

**Table 7:** Midgut heme transporter candidates selected for RNAi knockdown in adult *Ae. aegypti*

Gene ID	log2FC	FDR	Annotation	# TM
AAEL000417	−0.58947	0.02177	monocarboxylate transporter	12
AAEL000461	−0.59269	0.00551	protein_coding	3
AAEL001938	−0.85206	0.00624	ATP-binding cassette sub-family A member 3, putative	17
AAEL002557	−1.17180	9.42E-07	cationic amino acid transporter	12
AAEL003318	−0.54787	0.01182	oligopeptide transporter	10
AAEL004513	−0.75116	0.00589	neurotransmitter gated ion channel	3
AAEL009531	−0.65507	0.00053	niemann-pick C1	14
AAEL012440	−0.78354	4.60E-05	sodium-bile acid cotransporter	10

While knockdown of 4 of the 8 genes tested resulted in a significant decrease in ZnMP uptake, each one contributed less than a 35% reduction in ZnMP fluorescence. To test whether these genes contribute to heme import into the midgut epithelium through independent redundant systems, a mixture of dsRNAs targeting AAEL000417, AAEL003318, AAEL004513 and AAEL012440 was injected into female mosquitoes alongside an EGFP control dsRNA. However, when the level of RNAi-based knockdown was evaluated using RT-qPCR, significant reduction of transcripts was observed for only 2 of the 4 genes (AAEL003318 & AAEL012440) (Fig. 6d). Following dissection of the midguts 4 days later and ZnMP treatment, the ZnMP uptake was compared between the EGFP control and the AAEL003318/AAEL012440 dsRNA group. Surprisingly, no difference in ZnMP uptake was observed between the candidate dsRNA group and the control (*p*-value = 0.3451) (Fig. 6c). As heme is an essential signal in vitellogenesis, we reasoned that decreased heme uptake might result in delayed ovarian growth following a bloodmeal. Ovaries were dissected from mosquitoes injected with the dsRNA mixture at 12 h (Fig. 6e) and 24 h (Fig. 6f) post blood feed. Consistent with our expectation, ovary size was significantly smaller in the experimental group at 12 h after blood feeding (*p*-value = 0.0191). However, this difference was not observed at the 24-h time point (*p*-value = 0.2723), indicating that any delay in vitellogenesis was only temporary.

## Discussion

Despite the importance of heme as a functional group in many proteins, its use as a signaling molecule, and its role as a nutrient in *Ae. aegypti*, intracellular heme trafficking is not well understood in this mosquito species. Dietary heme from a bloodmeal, but not synthesized heme, also serves as a signal for vitellogenesis [12–16]. Therefore, we conducted transcriptomic analyses of *Ae. aegypti* midguts and cell lines to identify genes whose expression was altered upon heme overexposure or deficiency conditions with the goal to identify genes that encode transporters of heme across cellular membranes, like those found in *C. elegans* and *R. microplus* [26, 35]. Our differential expression and soft cluster analyses indicated that mosquito cell lines alter the expression of many genes in response to heme deficiency. Surprisingly, only a small number of multipass transmembrane domain containing DEG were both highly differentially expressed (2-fold/down) and displayed one of the hypothesized expression patterns for genes with heme import (↑ in heme deficiency & ↓ in heme exposure) or export functions (↓ in heme deficiency & ↑ in heme exposure) in each dataset. However, when these candidate lists were cross-compared between DEG experiments, there was little to no overlap. This failure to identity one or more strongly differentially expressed multipass transmembrane protein across the cell lines and conditions examined suggests the possibility that each cell line could have a redundant system of heme import, whereby multiple transport proteins each contribute to the total heme accumulation in the cell. Alternatively, it is possible that these cells lines regulate heme transport translationally or post-translationally, and thus differences would not be measurable in these analyses.

Transcriptomic changes in response to heme-exposure in Aag2 cells [27] and blood feeding in adult females [30] have been explored previously. When the multipass transmembrane domain containing genes present in these analyses were compared to the DEGs and the genes present in the import-like/export-like clusters identified in our cultured cell analyses, a small number of genes were found in common between them. Bottino-Rojas et al. (2015) exposed Aag2 cells to 50 μM heme for 24 h, resulting in 28 DEG (≥2TM) with predicted functions related to transport and fold changes greater than 2-fold. However, they tested a higher heme concentration for a shorter incubation time than what was explored in this set of experiments. As the majority of the 28 DEG were also found in at least one of our cultured cell heme-exposure experiments (78.6%), similar differential expression patterns can be inferred despite both the differing experimental heme incubation times and concentrations performed. Additionally, our analyses led to the identification of another 249 genes (2-fold/down, ≥2TM) with predicted functions related to transport across all our experimental conditions not previously identified by Bottino-Rojas et al. Bonizzoni et al. (2011) explored the transcriptomic changes induced by a bloodmeal in adult females 5 h post feeding and found < 400 highly expressed DEG with 2+ TM domains. Of these, 46% of these were found either differentially expressed or present in import-like or export-like clusters in our cultured cell analyses. In addition, only 3.9% of all TM containing DEG present in bloodfed females were also differentially expressed in heme-exposed midguts. This disimilarity between the datasets could be due to differences in cell/tissue (Whole female carcass vs cultured cells/midgut tissue) examined or particular heme-treatment performed (whole vertebrate blood vs heme alone). As Bonizzoni et al. (2011) performed their transcriptomic analysis with the whole female mosquito, they emphasized those DE genes found across the carcass, not those specific to the midgut epithelium.

Our transcriptomic analysis of heme-exposed midguts resulted in 243 DEG, of which 40 were identified as candidates for genes with potential heme transport function based on the prediction of 2+ transmembrane domains and predicted functions either related to transport or were unknown. Out of the 243 DEGs we noted that 11 highly upregulated genes in response to heme-exposure were of mitochondrial origin. Whether this is due to specific modulation of these genes or general proliferation of the mitochondria in response to heme is unclear, however changes in mitochondrial biogenesis could be verified through measurement of the ratio of mitochondrial and nuclear-encoded proteins [36, 37]. Overall we observed low differential expression in our ex vivo midgut transcriptomic analysis which could be due to the high variability observed between all of the heme replicate samples (Fig. S14). This extra source of variability in the dataset could have reduced the statistical power of the DE analysis, overall lowering the height of the expression changes observed. This variability could potentially be reduced if heme-exposure was examined at an earlier timepoint to limit the time spent in the cultured medium prior to measurement.

As we were most interested in identifying membrane bound heme transporters either exclusively present in midgut epithelial cells, or present in multiple tissues/life stages including the adult female midgut, we performed RNAi knockdown of a subset of the 40 genes in both cultured cells and midgut epithelial cells. Across the two cultured cell knockdown experiments, only AAEL008664 showed significantly increased ZnMP uptake compared to the EGFP control. However, the increase in fluorescence observed, 28.8%, was modest, suggesting that more than one gene product might be responsible for heme transport into and out of the cell. When an RNAi knockdown screen was performed on adult female midguts, 4 genes were identified with putative functions in heme import which showed a modest reduction in ZnMP uptake of > 20%. Additionally, the fact that only modest ZnMP fluorescence changes were observed post knockdown could be due to membrane-bound proteins having a lower average protein turnover rate than soluble ones [38, 39]. To test the hypothesis that multiple genes may be involved in heme transport, 2 genes were successfully knocked down simultaneously (AAEL003318 and AAEL012440), however the decreased fluorescence observed previously was not reproduced. Both genes were also observed as highly upregulated in the previous differential expression analysis of 5-h post blood feeding adult females vs sugar fed (AAEL003318: ~ 18-fold increase; AAEL012440: ~ 2.08-fold increase), implying a role in blood digestion [30]. In addition, a sodium bile acid cotransporter (IrSigP-109,984), orthologous to AAEL012440 (through reciprocal best match), was implicated in blood digestion in *Ixodes ricinus* ticks, which could indicate a convergent regulatory mechanism across blood-feeders [40].

Direct orthologues of characterized vertebrate heme transport proteins [41–44] were not found in *Ae. aegypti* [25]. This informed our decision to perform de novo identification and characterization of novel transporters through examination of transcriptomic changes observed due to heme deficiency and overexposure conditions. However, after the start of this project, a novel heme transport protein, ABCB10, was characterized in the tick *R. microplus* [35, 45]. Like *C. elegans, R. microplus* lacks a functional heme biosynthesis pathway [35, 46]. An orthologue to ABCB10, AAEL000434, is present in *Ae. aegypti*, however it was not differentially expressed in any of the analyses performed in this study.

It is possible that regulation of heme transport occurs, not transcriptionally as was tested in the analyses described in this study, but rather translationally or post-translationally. This hypothesis could be tested through a proteomic analysis at various time points post heme exposure, though such an analysis would be limited to the detection of overall changes in protein abundance and thus could not distinguish all potential protein regulatory changes. A proteomic analysis was recently performed in this mosquito targeting the peritrophic matrix and its heme binding proteins [34], the protocol of which could be adapted here to examine midgut epithelial cells instead. Otherwise, gain of function studies could be performed in a model organism such as yeast [47] or *C. elegans* that has been modified to temporarily remove native heme import functionality [48]. Yeast preferentially uptake iron to synthesize their own heme rather than scavenging exogenous heme [49]; however, a separate heme uptake system is activated when heme biosynthesis is compromised. As a result, strains deficient in the FET3 protein (Ferroxidase component of Fe transport complex) could serve as a recipient for potential heme/iron transporters from *Ae. aegypti*. Despite the potential of this system to characterize heme transport proteins, the rescue of heme import in a model organism would be dependent on proper exogenous protein folding as well as the transport protein’s ability to function as a homo-oligomer. Lastly, while this study has provided evidence against one or a few highly differentially regulated genes involved in heme transport in the *Ae. aegypti* mosquito, much work remains to pin down what type of regulation is responsible for changes in heme uptake and which genes are directly responsible for heme transport during blood digestion.

## Conclusions

In this study, we examined the response of two *Ae. aegypti* cultured cell lines and midgut epithelial tissue to heme overload or deficiency conditions in order to identify novel genes involved in heme transport as no genes with heme transport capacity have been identified in this mosquito previously. Both of the cultured cell types appear to upregulate heme import in heme deficiency conditions, while in heme-exposed midguts, a down-regulation of heme import in response to heme overload was observed. Transcriptomic analyses did confirm differential expression in response to heme deficiency and heme overload in cultured cells. However, despite observing many DEG between the heme treatments in each analysis, no strong candidates for genes critical for heme transport were found. This could indicate that either the cells examined have a redundant system of heme import with the total heme accumulation in each cell due to multiple transport proteins or that these cells do not regulate heme transport transcriptionally. In the end, only one potential membrane bound heme transport gene, AAEL008664 was identified through the RNAi screen in Aag2 cultured cells, a protein of unknown function, although further work is necessary to identify its cellular location, and specific function. In the adult RNAi screen, four genes were initially identified with potential roles in heme import due to modest decreases in ZnMP fluorescence. However, upon simultaneous knockdown of two of these genes, AAEL003318 and AAEL012440, the resulting fluorescence failed to indicate a corresponding change in the heme import pathway suggesting more work is needed to elucidate the specific functions of these genes. In conclusion, the knowledge provided by this study yields valuable insight into the regulatory responses initiated during heme deficiency and overload conditions and the membrane bound heme transport system in *Ae. aegypti*.

## Methods

### Cultured cell growth conditions

*Ae. aegypti* A20 and Aag2 cells were maintained in Leibovitz’s L-15 media (Gibco) supplemented with 10% FBS (Atlanta Biologicals) and 1% Pen-strep (Corning) at 28 °C. Aag2 cells were also maintained in Schneider’s *Drosophila* Media (Thermo Fisher) with 10% FBS (Atlanta Biologicals) and 1% Pen-strep (Corning) at 28 °C. Both cell lines are available upon request.

### Mosquito rearing

For all experiments, *Aedes aegypti* (Liverpool strain; obtained from Virgina Tech) was reared at 28 °C in humidified growth chambers at 70% RH and a day: night cycle of 14H:10H. The mosquito colony was maintained on defibrinated sheep blood (Colorado Serum Company, CO). Females 1–3 days old were selected for all experiments. All mosquitoes were fed a 10% sucrose solution with 1% Penicillin-streptomycin 2 days prior to dissection and a 10% sucrose solution prior to dsRNA injection. All females were starved of 10% sucrose 12 h prior to blood feeding.

### Qualitative assay of ZnMP uptake in mosquito cultured cells

Zinc Mesoporphyrin (ZnMP) (Frontier Scientific, Logan, UT) stock solution (0.3 mM) was made following a previously described protocol [50]. Following filter sterilization (pore diameter 0.22 μm); the solution was aliquoted and stored at -20 °C until needed. Cultured cells (Aag2 in Schneider’s *Drosophila* media) seeded onto 6-well plates were incubated for 72 h in normal growth media, media + 0 μM heme (heme-deficient) or media + 10 μM heme (heme-overexposure). All subsequent steps were performed in a cell culture hood in the presence of minimal light [0.4 lm/ft^2^ on average as measured with a HOBO data logger (Part #U12–012; Onset; Bourne, MA)] to prevent degradation of the ZnMP. ZnMP uptake was performed (0, 5 & 10 μM) for 30 min in uptake buffer following a procedure adapted from Worthington et al., 2001. The uptake buffer used during the incubation and wash procedures consisted of 50 mM HEPES, pH 7.4, 130 mM NaCl, 10 mM KCl, 1 mM CaCl_2_ and 1 mM MgSO_4_. Following the incubation, the cells were washed once with uptake buffer + 5% BSA, then 2x with uptake buffer. Cells were imaged in 1x PBS at 100x magnification using the DsRED filter or the propidium iodide (PI) filter of the EVOS FLoid Cell Imaging Station (Thermo Fisher) or the Zeiss Axio Observer Microscope (Carl Zeiss Microscopy, Thornwood NY).

### Quantitative ZnMP assay in mosquito cultured cells

Aag2 cells incubated for 48 h in normal growth media, heme deficient media and heme overexposure media, were harvested and reseeded onto a 96-well black clear bottomed BRANDplate® Cell grade Premium (Brand Wertheim, Germany) at 2 × 10^6^ cells/mL per well and 32 wells per treatment type. Twenty-four hours later, the 96 well plate was incubated with 5 μM ZnMP (100 μL/well) for 30 min at 28 °C. The same wash steps were performed as previously described. Next, 2.5 μM Calcein AM (Thermo Fisher) diluted in 1xPBS was added to each well and incubated at 28 °C for 30 min. The average of 21 fluorescence measurements taken across each well at Ex. 540/Em. 580 using a Spectramax i3x (Monochromator, Fluorescent & Well Scan options selected) was obtained. The number of cells present in each well was determined by Calcein fluorescence using a MiniMax 300 (MiniMax, Imaging & Endpoint options selected) (Molecular Devices, San Jose CA) using the green channel (Ex. 456/Em. 541). A total of 4 photos (5 ms exposure & 120 μm focus adjustment) were taken per well centering around the middle of each well. The number of cells per well was counted by using the discrete object analysis in the SoftMax® Pro Software MiniMax Imaging Edition v6.5.1 to identify cells based on the size and shape of their green fluorescence. The ‘draw on image’ option was selected to allow customized selection of cells during the counting procedure. The ZnMP fluorescence per cell was calculated by dividing the mean fluorescence per well by the number of cells counted for each well. As the data failed the D’Agostino-Pearson omnibus normality test, the mean ZnMP fluorescence/cell for each treatment was compared using the Kruskal-Wallis and Dunn’s multiple comparisons tests as performed by GraphPad Prism v7.02 (San Diego, CA).

### Quantitative ZnMP and pHrodo™ green dextran assays of ex vivo *Ae. aegypti* midguts

In the initial ex vivo experiment, midguts from 3 to 5 day old sugar fed female *A. aegypti* (*n* = 60) were dissected in 1x PBS, transferred to a biosafety cabinet and split evenly between 3 treatments in 6-well culture plates (2 mL media/well). Midguts were subject to one of the following treatments: Schneider’s *Drosophila* Media (Thermo Fisher) with 10% FBS (Atlanta Biologicals) and 1% Pen-strep (Corning) (FBS), Schneider’s *Drosophila* Media + 1% Pen-strep (0 μM Heme) and Schneider’s *Drosophila* Media + 1% Pen-strep + 10 μM Hemin Chloride (Sigma-Aldrich) (10 μM Heme).

All midgut samples were incubated at 28 °C for 35 h with midguts transferred into fresh media on a new plate every 8–12 h. Following the media incubation, the midguts were incubated with 10 μM ZnMP for 30 min at 28 °C or with 100 μg/mL pHrodo™ green dextran (Thermo Fisher Scientific, Waltham MA) for 20 min at 37 °C. Photos were taken using the DsRED (ZnMP) or GFP filter (Dextran) of the Leica M165 FC stereomicroscope equipped with a DFC3000C digital camera at 16x magnification. Photos of 0 μM ZnMP/Dextran-treated midguts were taken prior to fluorophore incubation from a subset of the midguts in each treatment group. The resulting images were analyzed by ImageJ [51] to determine mean pixel intensity per midgut background corrected to remove channel and tissue auto fluorescence. Means were either compared using a one-way ANOVA with individual means compared using Tukey’s multiple comparisons test or an unpaired two-tailed t-test as performed by GraphPad Prism v7.02 (San Diego, CA). If the data failed the D’Agostino-Pearson omnibus normality test, Kruskal-Wallis test and Dunn’s multiple comparisons test were performed instead.

### Heme treatments and sample preparation for RNA-seq of both cultured cells and midgut tissue

Aag2 and A20 cultured cells were exposed to heme deficiency and overexposure conditions across 4 different experiments as described in Table 1. All 4 experiments had 3 biological replicates performed per heme treatment. Heme exposed midgut samples were also prepared from dissected midguts (3–5-day sugar fed females) incubated in the 0 μM Heme and 10 μM heme treatments previously described. A total of 4 biological replicates were performed with 30 pooled midguts per replicate which were snap frozen in liquid nitrogen at the end of the incubation period. For all samples, RNA was isolated using the TRIzol reagent (Thermo Fisher), following the manufacturer’s protocol. The resulting RNA was DNase treated (DNase I: New England Biolabs) and then quantified using a Nanodrop (Thermo Fisher). One microgram of total RNA of each sample was prepared for sequencing using the TruSeq RNA v2 Illumina library preparation kit (Illumina San Diego, CA) or the NEBNext Ultra RNA library preparation kit (New England Biolabs Ipswich, MA). Both protocols utilized sequential first and second strand cDNA synthesis with random priming and PCR library enrichment of 10 cycles.

### Sequencing & Computational Analysis of Heme-exposed cultured cells and Midguts

Paired-end mRNA sequencing with 150 bp read length was performed on all cultured cell samples on an Illumina HiSeq 2500 (Genomics Sequencing Center at the Virginia Biocomplexity Institute). Single-end sequencing with 75 bp read length was performed on all heme-exposed midgut samples on an Illumina Nextseq 500 (Texas A&M’s Institute for Genome Sciences and Society). Reads were aligned to the *Ae. aegypti* reference genome [AaegL5, obtained from www.vectorbase.org [38]] using HISAT2 v2.1.0 with default parameters [52] through Texas A&M’s High Performance Research Computing Ada server. Mapped reads were sorted and reads with low quality mapping scores were removed (−q10) with SAMtools view and SAMtools sort v1.7. The number of reads per mRNA transcript was calculated with the BEDtools suite v2.19.1. Differential expression was determined using the exact test [53] as implemented in EdgeR [54] at a false discovery rate (FDR) of 0.0001. FDR was calculated using the ‘topTags’ function with the default *p*-value correction method ‘BH’ [55]. Transmembrane domain predictions of the DEGs were determined by the TOPCONS webserver [56]. The number of TM domain containing genes in a random subset of genes from the *Ae. aegypti* genome (*n* = # of DEG in the comparison tested) was determined using custom scripts (additional file 5). The mean number and standard deviation of 30 random samples was calculated for each of the differential expression analyses. Finally, a two-tailed z-test was performed for each comparison tested to determine whether the number of TM containing genes found in the analyses were significantly distinct from those found by chance alone.

Soft cluster analysis was performed on all cultured cell datasets using the Mfuzz R package [57, 58]. Default parameters were followed except for the following: the optimal fuzzifier value, m, was determined using the mestimate function and the optimal number of clusters were determined using the cselection function. All four datasets were also compared by using 4-way Venn diagrams, comparing the import-like clusters and the export-like clusters between the datasets [59].

### Reverse genetic experiments in *Aedes aegypti* cultured cells and adult females

#### dsRNA production

PCR amplicons of each candidate heme importer were generated using Phusion polymerase (New England Biolabs Ipswich, MA), primers listed in Supplemental Table 6 and cDNA generated from female *Ae. aegypti* Liverpool strain mosquitoes as template. The EGFP amplicon was based off a GFP containing plasmid, Ac5-STABLE1-neo a gift from Rosa Barrio & James Sutherland (Addgene plasmid #32425; http://n2t.net/addgene:32425; RRID:Addgene_32,425) [60]. An additional set of primers targeting different regions of the genes AAEL001495, AAEL004657, AAEL008664, AAEL012226, AAEL012440 were designed as well. The MEGASCRIPT RNAi kit (Thermo Fisher) was used to make the dsRNA with T7 polymerase enzyme making both strands during the same 5-h incubation at 37 °C. The resulting dsRNA was nuclease treated to remove any ssRNA and dsDNA template remaining in the sample followed by purification using the MEGACLEAR kit (Thermo Fisher).

#### Transfection of cultured cells

dsRNA transfections were performed using Effectene (Qiagen Hilden, Germany) following the standard transfection protocol with cells at 70–80% confluency. Specifically, 2.5 μg of dsRNA, 8 μL Enhancer, 129.5 μL Buffer EC and 25 μL Effectene was used for each well of a 6-well plate. A control dsRNA targeting GFP was performed with each plate. At 24 h post transfection, the media was replaced in each well with 2 mL Schneider’s *Drosophila* media + 1% Pen-strep (Heme-deficient). At 3 days post transfection, the cells were harvested and reseeded at 2 × 10^6^ cells/mL per well with a total volume in each well of 100 μL into a 96-well black clear bottomed BRANDplate® Cell grade Premium (Brand Wertheim, Germany) 12 wells per dsRNA, with any remaining cells pelleted by centrifugation at 4 °C and frozen at -80 °C after discarding the supernatant. Transfected cells seeded in 96 well plates were incubated with 5 μM ZnMP for 30 min at 28 °C, 100 μL solution per well, 24 h later. The ZnMP and Calcein incubation, wash procedures and fluorescent imaging were performed as described above.

#### Statistical analysis in cultured cells

The ZnMP fluorescence per cell values were then normalized to the exogenous GFP control dsRNA values. The lme4 package in the R statistical software was used to run a linear mixed-effects model analysis [61]. GFP was set as the reference for the analysis using the relevel command. The hypothesized formula for the model is given below with the relationship between cell fluorescence and dsRNA gene knockdown accounting for the potential random effects due to the day each experiment was performed.

$$ Fluorescence\sim Gene+\left(1| Day\right)+\varepsilon $$

Finally, all data for each replicate was pooled and graphed using GraphPad Prism v7.02 (San Diego, CA).

#### Injection

A total of 400 ng dsRNA was injected into each adult female *Ae. aegypti* using a Nanoject III microinjector (Drummond) with a glass needle drawn from a capillary. For each dsRNA, 3 replicates were performed with ~ 30 females per replicate. A pooled dsRNA group was also performed (400 ng/dsRNA) with the genes AAEL000417, AAEL003318, AAEL004513 and AAEL012440 except each replicate was ~ 90 females. Pooling occurred directly following quantification of newly prepared dsRNA, adding 400 ng of each dsRNA per mosquito to be injected to a microcentrifuge tube, followed by freezing aliquots at -80 °C until the day of injection. A control group of females was injected for each experiment with dsRNA targeting EGFP.

#### Dissection, ZnMP incubation and fluorescence measurement in adults

At 3 days post injection, ~ 30 midguts were dissected from the surviving females in 1x PBS and placed into 1.5 mL microcentrifuge tubes. A 30-min incubation in 10 μM ZnMP at room temperature was performed in aluminum foil covered microcentrifuge tubes placed on a tube rotator (VWR). Once the ZnMP incubation and wash steps were finished, treated midguts were placed on microscope slides in 1xPBS sectioned off with an ImmEdge™ hydrophobic barrier pen (Vector Laboratories). Images were taken of each midgut at 16x magnification with the DsRED filter of the Leica M165 FC stereomicroscope equipped with a DFC3000C digital camera. The resulting images were analyzed by ImageJ [51].

#### Statistical analysis of adult midguts

The mean pixel intensity values were normalized to the EGFP control fluorescence values. A linear mixed-effects model analysis on the normalized values was performed using the lme4 package in the R statistical software [61]. GFP was set as the reference for the analysis using the relevel command. The hypothesized formula for the model is given below with the relationship between cell fluorescence and dsRNA gene knockdown accounting for the potential random effects due to the day each experiment was performed and area of a midgut.

$$ Fluorescence\sim Gene+\left(1+ Day| Area\right)+\varepsilon $$

Finally, all data for each replicate was pooled and graphed using GraphPad Prism v7.02 (San Diego, CA).

#### Blood feed of remaining females and 12- and 24-h dissection and ovary measurements

The remaining females from the pooled dsRNA group and its matching EGFP group were blood fed for 1 h at 9 am 3 days post injection. At 12- and 24-h post blood feeding ~ 30 females were dissected harvesting the midguts and the ovaries. Post removal of the meal for each midgut, the midguts were pooled in 50 μL 1x PBS for each group and frozen in liquid N_2_ before storage at -80 °C. Each pair of ovaries was photographed at 25x magnification with the MU300 AmScope microscope digital camera attached to a Leica M60 microscope (AmScope Irvine, CA; Leica, Germany) on a reticle stage micrometer microscope slide (Electron Microscopy Sciences, PA).

#### Ovary size calculation and statistical analysis

The resulting ovary images were analyzed by ImageJ [51] to measure the size of each ovary. Ovary size was scaled by using the 100 μm ruler present on the slide and normalized to the EGFP control ovary measurements. Finally, all data for each replicate was pooled and a two-tailed unpaired t-test performed using GraphPad Prism v7.02 (San Diego, CA).

### RT-qPCR validation of RNAi knockdown and RNA-seq

Quantitative real-time PCR (RT-qPCR) was performed on a CFX69 Touch Real-Time PCR Detection System with SsoAdvanced Universal SYBR Green Supermix (Bio-Rad, CA). Primers were designed using the Primer3 server (version 4.1.0) aiming for sequence length of 80-150 bp with DNA hairpin folding accounted for using mfold with the folding temperature set to 60 °C and Mg^++^ concentration set to 1.5 mM [62, 63]. Each primer pair was tested with an annealing temperature gradient and pairs with a single peak in their melt curves at 59 or 61.4 °C were then empirically verified to have percent amplification efficiencies ranging from 90 to 110%. The gene rpS7, AAEL009496, was used as a reference housekeeping gene; the primer sequences, annealing temperature and primer efficiency for it and the other primers used are shown in Supplemental Table 6 (Additional file 2). The program run on the thermocycler was 95 °C for 30 s, followed by 39 cycles of 15 s at 95 °C and 30 s at 59 or 61.4 °C annealing temperature finishing with a melt curve of 65-95 °C measuring every 0.5 °C. For the genes involved in RNAi knockdown confirmation, the RT-qPCR results were normalized to the EGFP knockdown control samples and graphed using GraphPad Prism v7.02 (San Diego, CA). Statistical significance between the control EGFP and each gene knockdown was determined using an unpaired two-tailed t-test (*α* < 0.05). Otherwise, the RT-qPCR log2 relative quantification values were compared to the log2 fold change values obtained from the differential expression analysis using GraphPad Prism.

## Supplementary information

**Additional file 1 **RNA-seq_Supplement_tables. Table S1-S5: All RNA-seq transcriptomic analyses for all conditions examined. This file includes the full spreadsheet for the differential expression and soft cluster analyses of all genes examined. Table S1. Aag2 cells exposed to 10 μM Heme or 0 μM Heme in Schneider’s *Drosophila* growth media for 72-h. Table S2: Aag2 cells exposed to 20 μM Heme for 48-h. Table S3: A20 cells exposed to 10 μM Heme or 0 μM Heme for 72-h. Table S4: Aag2 cells exposed to 10 μM Heme or 0 μM Heme in Leibovitz’s L-15 growth media for 72-h. Table S5: *Ae. aegypti* midguts exposed to 10 μM Heme or 0 μM Heme in Schneider’s *Drosophila* growth media for 35-h.

**Additional file 2.** Table S6. Primers utilized for RT-qPCR and dsRNA production. This file includes the primer sequences for all primers utilized in the Real Time quantitative-PCR and dsRNA production described in this paper.

**Additional file 3.** Supplemental_Text. This file includes the supplemental text describing the details of the additional cultured cell experiments performed in Aag2 cells and A20 cells.

**Additional file 4 Fig. S1**. Treatment conditions of A20 and Aag2 mosquito cells prior to RNAseq analysis. Schematic representation of the various treatments used to prepare samples for RNAseq. Cell type (Aag2/A20), incubation time (48 h, 72 h), growth media type (L-15, Schneider's Drosophila), and heme supplement (0 μM, 10 μM, 20 μM), with (Normal media, indicated by 50 mL conical tube) or without (indicated by mini centrifuge tube) FBS present in the media. Schematic was generated using Biorender through a license from Texas A&M University. **Fig. S2**. Multidimensional Scaling Plot of RNAseq data derived from Aag2 cultured cells grown in Schneider’s *Drosophila* medium. Multidimensional scaling plot displaying transcriptomic changes in Aag2 cells grown in Schneider’s *Drosophila* medium exposed to heme overload or heme deficiency conditions. The cells grown in normal growth media are circled in blue (FBS), the cells exposed to heme overload are circled in green (10 μM Heme) and the cells exposed to heme deficiency are circled in orange (0 μM Heme). **Fig. S3.** RNAseq-based transcriptomic analyses after 48-h heme treatment in Aag2 cells. (**A**) Multidimensional scaling plot. The FBS treated group is circled in blue and the FBS + 20 μM heme group is circled in green. (**B**) Log2 fold change (logFC) vs Log10 counts per million (logCPM) plots of expressed genes; genes with an adjusted *p*-value corrected for multiple testing (False discovery rate: FDR) of < 0.0001 are shown in red. (**C**) Volcano plot of genes expressed in analysis. (**D**) 6 distinct gene expression profile clusters were identified through soft clustering. Only those genes with membership scores > 50% are shown in each cluster. Red lines show high membership in a cluster while blue indicates low membership. Heme import-like clusters are highlighted in red and export-like clusters in blue. The different heme treatment groups are given on the X-axis of each cluster; n, the number of genes present in a cluster. **Fig. S4**. Multidimensional Scaling Plot of RNAseq data derived from A20 cells grown in normal, heme-deficient or heme overload media. Cells grown in normal growth media are circled in blue (FBS), the cells exposed to heme overload are circled in green (10 μM Heme) and the cells exposed to heme deficiency are circled in orange (0 μM Heme). **Fig. S5**. Multidimensional Scaling Plot of RNAseq data derived from Aag2 cultured cells grown in L-15 media. Multidimensional scaling plot displaying transcriptomic changes in Aag2 cells grown in L-15 media exposed to heme overload or heme deficiency conditions. The cells grown in normal growth media are circled in blue (FBS), the cells exposed to heme overload are circled in green (10 μM Heme) and the cells exposed to heme deficiency are circled in orange (0 μM Heme). **Fig. S6**. RNAseq-based transcriptomic analysis after 72-h heme treatment in A20 cells. A20 cells grown with FBS, 0 μM heme and 10 μM heme (**A-C**) Log2 fold change (LogFC) vs Log10 counts per million (LogCPM) graph of genes expressed in analysis for each comparison. Genes with an adjusted *p*-value corrected for multiple testing (False discovery rate: FDR) of < 0.0001 are shown in red. (**D**-**F**) Volcano plot of genes expressed in analysis. (**A**&**D**) FBS vs 10 μM heme (**B**&**E**) FBS vs 0 μM heme (**C**&**F**) 0 μM heme vs 10 μM heme. (**G**) 15 gene expression profile clusters were identified through soft clustering. All genes retained in a cluster scored > 50% membership to that cluster. Red lines indicate high membership values in the cluster, blue indicates low membership. The different heme treatment groups are given on the X-axis of each cluster. Heme import-like clusters are highlighted in red and export-like clusters are highlighted in blue; n, the number of genes present in a cluster. **Fig. S7.** RNAseq-based transcriptome analysis after 72-h heme treatment of Aag2 cells grown in L-15 media. Aag2 cells grown with FBS, with 0 μM heme or with 10 μM heme (**A-C**) Log2 fold change (LogFC) vs Log10 counts per million (LogCPM) graph of genes expressed for each comparison. Genes with an adjusted *p*-value corrected for multiple testing of < 0.0001 are shown in red. (**D-F**) Volcano plot of genes expressed in analysis. (**A**&**D**) FBS vs 0 μM heme (**B**&**E**) FBS vs 10 μM heme (**C**&**F**) 0 μM heme vs 10 μM heme. (**G**) 15 gene expression profile clusters were identified through soft clustering. Only genes with membership scores of > 50% are shown in each cluster. Red lines indicate high membership values in the cluster, blue indicates low membership. Heme import-like clusters are highlighted in red and export-like clusters are highlighted in blue. The different heme treatment groups are given on the X-axis of each cluster; n, the number of genes present in a cluster. **Fig. S8**. TM containing genes shared between the differential expression analysis and the cluster analysis of Aag2 cells treated with heme for 72 h in Schneider’s *Drosophila* media. Transmembrane domain containing genes found significantly expressed in DE analysis were compared to those identified in the cluster analysis. (**A**) Downregulated genes in the absence of heme vs Upregulated genes in the presence of heme vs those in import-like clusters. (**B**) Downregulated genes in the absence of heme vs Upregulated genes in the presence of heme vs those found in export-like clusters. **Fig. S9**. TM containing genes shared between the differential expression analysis and the cluster analysis of Aag2 cells treated with heme for 48 h. Transmembrane domain containing genes found significantly expressed in DE analysis were compared to those identified in the cluster analysis. (**A**) Upregulated genes in the presence of heme vs those in import-like clusters. (**B**) Downregulated genes in the presence of heme vs those found in export-like clusters. **Fig. S10**: TM containing genes shared between the differential expression analysis and the cluster analysis of A20 cells treated with heme for 72 h. Transmembrane domain containing genes found significantly expressed in DE analysis were compared to those identified in the cluster analysis. (**A**) Downregulated genes in the absence of heme vs Upregulated genes in the presence of heme vs those in import-like clusters. (**B**) Downregulated genes in the absence of heme vs Upregulated genes in the presence of heme vs those found in export-like clusters. **Fig. S11**. TM containing genes shared between the differential expression analysis and the cluster analysis of Aag2 cells treated with heme for 72 h in Leibovitz’s L-15 media. Transmembrane domain containing genes found significantly expressed in DE analysis were compared to those identified in the cluster analysis. (**A**) Downregulated genes in the absence of heme vs Upregulated genes in the presence of heme vs those in import-like clusters. (**B**) Downregulated genes in the absence of heme vs Upregulated genes in the presence of heme vs those found in export-like clusters. **Fig. S12**. Potential Heme Importers and Exporters found in independent RNA-seq experiments after treatment with Heme. Candidate genes were chosen in each heme exposed cultured cell dataset based on expression pattern and having at least one transmembrane domain prediction. Expression patterns expected for potential transcriptionally regulated exporters (**A**) or importers (**B**). **Fig. S13**: Heme treatment reduces ZnMP uptake in *Aedes aegypti* female midguts at multiple heme concentrations. *Ae. aegypti* female midguts were incubated in varying concentrations of heme ranging from 0 μM to 10 μM. Photos for each heme concentration taken before (**A**) or after (**B**) ZnMP incubation. Raw fluorescence intensity (**C**) or background corrected (**D**) measurements of each midgut. Red-filled points correspond to the matching photo given in (**A**) or (**B**). WL = White Light. **Fig. S14**. Multidimensional scaling plot of RNAseq data derived from heme treated midguts. Multidimensional scaling plots displaying transcriptomic changes in dissected *Ae. aegypti* midguts exposed to heme overload or heme deficiency conditions. The midgut replicates exposed to heme overload are circled in green (10 μM Heme) and the midgut replicates exposed to heme deficiency are circled in orange (0 μM Heme). (**A**) MDS plot containing all 4 replicates of both heme treatments. 2 samples do not cluster with their other replicates, 0 μM replicate 1 and 10 μM replicate 2, circled in red. (**B**) MDS plot containing 3 replicates per heme treatment as the 2 samples that failed to cluster with their groups were removed.

**Additional file 5 **Supplemental code file. This file contains an example set of Linux commands used to perform random sampling of *Ae. aegypti* genome for genes encoding proteins with transmembrane domain predictions

## Data Availability

The datasets generated and/or analyzed during the current study are available in the GEO repository, under the accession numbers: GSE141651 (Cultured cell datasets) and GSE140752 (Midgut). A20 and Aag2 cell lines, as well as LVP strain *Ae. aegypti* mosquitoes are available upon request. *Ae. aegypti* genome data is available at www.vectorbase.org.

## Abbreviations

*AeIMUCI*: *Ae. aegypti* intestinal mucin 1
BV: Biliverdin
ZnMP: Zinc Mesoporphyrin
FBS: Fetal bovine serum
DEG: Differentially expressed genes
TM: Transmembrane
ROS: Reactive oxygen species
logFC: Log2 fold change
logCPM: Log10 counts per million
RQ: Relative quantification
RT-qPCR: Real-time quantitative polymerase chain reaction
dsRNA: Double-stranded RNA
