# Supplementary material for: Transcriptomic analyses of Aedes aegypti cultured cells and ex vivo midguts in response to an excess or deficiency of heme: a quest for transcriptionally-regulated heme transporters
Source: BMC Genomics. 2020 Aug 31;21:604. doi: 10.1186/s12864-020-06981-5 (PMC7460771; doi:10.1186/s12864-020-06981-5)
Supplement: Supplementary file 3 — Additional file 3. Supplemental_Text. This file includes the supplemental text describing the details of the additional cultured cell experiments performed in Aag2 cells and A20 cells. [file 12864_2020_6981_MOESM3_ESM.docx]

**Supplemental Text**

*20µM heme-exposed Aag2 cells*

. Following RNAseq analysis, we determined that 8196 of the 19712 annotated transcripts in the *Ae. aegypti* mosquito were expressed across the cells grown in regular media and those grown in regular media with added 20µM heme, with biological replicates clustering together and treatment groups distinctly separate (Fig. S2A). Out of these, we found 551 significantly differentially expressed genes (Table S2, q-value <0.0001) of which 269 were upregulated and 282 downregulated during heme overexposure (Fig. S2B&C). As we were primarily interested in genes that encode proteins that might play a role in heme shuttling between the extracellular and intracellular space, we noted that 211 out of the 551 genes (41.3%) had at least 1 predicted transmembrane domain (TM), 106 which were upregulated and 105 downregulated in the presence of heme. The number genes with predicted TM domains was significantly more than what was expected by chance alone (obs 211. exp 175; p-value=0.001248). One of the genes found most highly upregulated was a cysteine synthase, AAEL008467 ( ~140-fold increase). This upregulation during heme overexposure conditions could indicate the presence of a high level of oxidative stress in these cells as the availability of cysteine is a limiting factor in the synthesis of the antioxidant glutathione. In addition, the reduced expression of AAEL004870 (~3.56 fold decrease), a cytochrome p450, during heme overload could indicate how unfavorable reactive oxygen species (ROS) generation is during high amounts of heme exposure as free heme molecules would generate an abundance of ROS. Overall, just 28 genes were a predicted to encode a protein with 2 or more TM domains and had an expression change in response to heme of over 2-fold up (19 genes) or down (9 genes). Only 2 of these genes however had over 4-fold expression changes, AAEL014246 (4.22 log2, ~18.7-fold change) a glucosyl/glucuronosyl transferase and AAEL006113 (2.93, ~7.6 fold change) a cystinosin. The predicted function of both genes would indicate their role in the heme stress response as they are involved in the removal of toxins and endogenous substances or mitigation of heme generated ROS through import of cystine into the cytosol, respectively. Of the 28 genes identified only one, AAEL014762, has been previously characterized as an iron-transporter in both *Ae. aegypti* and *D. melanogaster* (1, 2). When the predicted functions of the remaining 25 genes were examined only 7 had functions related to transport and 8 had no known predicted function.

We also performed a cluster analysis to identify those genes displaying an expression pattern consistent with that of heme import (upregulated upon heme deficiency) or heme export proteins (downregulated upon heme deficiency). In total, 3247 genes were mapped to 6 different expression clusters through a soft cluster analysis with each gene passing a membership score of ≥50% (Fig. S2D, Table S2). Of the 6 clusters, 2 showed the expected expression pattern of genes encoding heme import (Clusters 3 & 4) and 2 showed the expected expression pattern of genes encoding heme export (Clusters 5 & 6). Of the genes identified in the import-like and export-like clusters, 455 import-like genes (272 & 183 respectively) and 385 export-like genes (71 & 314 respectively) contained 1 or more predicted TM domains. The remaining 2 clusters (Clusters 1 & 2) contained genes that have expression patterns that match replicates of the opposite treatment type indicating those genes that do not appear to be regulated by heme exposure.

As two types of analyses were performed, we cross-compared the genes identified as DEG to those found in the import-like and export-like clusters. Of the 105 DE transmembrane domain containing genes that were downregulated in the presence of heme, the majority, 93.3% (n=98) were also found in the import-like clusters of the soft cluster analysis (n=468) (Fig. S8A). The same was true for the upregulated DE genes with TM domain predictions, 97.1% (103/106), were also found in the export-like clusters (n=401) (Fig. S8B). As a result, we determined that a large portion of the differentially expressed genes were also identified in the potential heme import and export expression clusters, with many more genes identified only in the clusters than the differential expression analysis (79% and 74.3% respectively). When the 28 genes previously identified in the DE analysis were examined, all 9 downregulated genes were found in the import-like clusters (Cluster 3: 7 & 4: 2) and all 19 upregulated genes were also identified in export-like cluster 6.

*Transcriptomic analysis of A20 cells in response to heme deficiency/excess*

After completing RNAseq and read mapping, we observed the expression of 8220 annotated transcripts across the 3 heme treatments in A20 cells, with the biological replicates of each treatment clustering together and each treatment distinctly separate (Fig. S3). Of these, 2815 DE genes were found significant in the normal media vs 10µM heme comparison (Fig. S5A,D), 745 DE genes from the normal media vs 0µM heme (Fig. S5B, E) and 3692 DE genes in the 0µM heme vs 10µM heme comparison (Fig. S5C, F) (Significance: q-value <0.0001; Table S3). Transmembrane domain prediction resulted in 658, 300 and 942 DE genes respectively with 1 or more TM domains. The number of TM domain containing genes found in each comparison was underrepresented (obs 658, exp 900; p-value <0.00001), enriched (obs 300, exp 240; p-value=0.00006) and underrepresented (obs 942, exp 1186; p-value<0.00001) respectively as compared to that expected by chance alone. Upon examination of the DE genes, we identified 6 multipass TM containing genes with decreased expression in heme deficiency conditions which may encode heme export proteins. These genes were downregulated in the absence of heme, upregulated in the presence of heme and were not significant in the normal media vs 10µM heme comparison. Otherwise, 92 multipass TM genes were found to match the expression pattern of potential heme import genes. These genes were upregulated in the absence of heme, downregulated in the presence of heme and not significant in the normal media vs 10µM heme comparison. Therefore, 98 genes were identified as candidates for further studies into heme transport across cellular membranes as they were differentially expressed in response to heme deficiency conditions.

Following the DE analysis, we performed a soft cluster analysis that assigned 6219 genes to one of 15 unique expression patterns that matched to a cluster ≥50% (Table S3). Genes that showed the pattern expected of potential heme import genes were found in Clusters 8 & 11 and those with the pattern of potential heme export genes were found in clusters 9 &13 (Fig. S5G). Of the genes present in the heme import-like clusters 448 had at least 1 predicted TM domain (219 & 229 respectively). Likewise, 143 of the genes in the heme export-like clusters had TM domain predictions (74 & 69 respectively). Additionally, gene expression changes in relation to nutrient levels can be observed in the cluster analysis (Clusters 1, 4 &15) as the media supplement, fetal bovine serum, is only present in the normal growth media. When we compare and contrast the TM containing DEGs to those present in the heme import-like and export-like expression pattern clusters many genes were shared between the 2 analyses. Overall, 68.5% of genes found downregulated in the presence of heme (0µM heme vs 10µM heme) and 49.3% of the genes found upregulated in the absence of heme (FBS vs 0µM heme) were also found in the import-like expression clusters (Fig. S9). Specifically, when the 92 highly differentially expressed genes identified as having expression patterns consistent with heme import in the differential expression analysis were compared to the heme import-like clusters, 79 were shared (Cluster 8: 8 & 11: 71) leaving 13 unique DEGs. Similarly, when the genes identified as having patterns consistent with heme export in the differential expression analysis were compared to those found in the export clusters 34.7% (FBS vs 0µM heme) and 25.2% (0µM heme vs 10µM heme) were shared between them. Of the 6 potential heme membrane bound exporter candidate genes identified in the differential expression analysis, 5 were also present in the heme export clusters in the soft cluster analysis (Cluster 9: 2 & 13: 3). Examination of the predicted functions of these 84 genes resulted in only 59 with either unknown or transport related functions. Therefore, if the results from both transcriptomic analyses are considered, we identified 55 genes and 4 genes with 2+ transmembrane domains with expression patterns consistent with the expectation for a role in heme import and heme export, respectively, and contained either unknown or transport related predicted protein functions when examining A20 cellular response to heme overload and deficiency conditions.

*RNAseq analysis of Aag2 cells to heme deficiency/excess in L-15 media*

In heme-exposed Aag2 cells grown in Leibovitz’s L15 media, 9484 annotated transcripts were expressed across the 3 heme treatments, with the biological replicates of each treatment type clustering together and the treatment groups separate (Fig. S4). Of these, 4166 genes were significantly differentially expressed when comparing normal media vs 0µM heme (Fig. S6A,D), 4979 genes in normal media vs 10µM heme (Fig. S6B,E) and 3200 genes in the 0µM heme vs 10µM heme (Fig. S6C,F) (Table S4; q-value <0.0001). Transmembrane domain predictions resulted in 1218, 1568 and 1121 genes respectively that contained at least 1 TM domain. The number of TM domain containing genes was underrepresented (obs 1218, exp 1339; p-value=0.00002), not significantly different (obs 1568, exp 1588; p-value=0.378) and overrepresented (obs 1121, exp 1019; p-value=0.00005), respectively, than that expected by chance alone. In total, we identified 7 genes that matched the expected heme-exporter expression pattern; these genes contained 2+ TM domains and were found downregulated in the absence of heme, upregulated in the presence of heme and were not significant in the normal media vs 10µM heme comparison. Otherwise, 6 genes were identified that matched the expected heme-importer expression pattern; These genes were predicted to have 2+ TM domains and were upregulated in the absence of heme, downregulated in the presence of heme and not significant in the normal media vs 10µM heme comparison. Therefore, 13 genes matching the expected expression pattern of genes that encode membrane bound heme transporter proteins were identified in this analysis.

To further explore the transcriptomic changes in the Aag2 cells treated with heme deficiency, we next examined the expression patterns across the heme treatments to identify those genes with a pattern of expression accordant with our expectations for heme transport proteins. In total, 7085 genes obtained a membership score of 50% and were assigned to one of 15 expression clusters (Table S4, Fig. S6G). Only 2 clusters showed the expression pattern expected for heme import genes, clusters 5 & 8, which contained 229 genes (123+106 respectively) with at least 1 transmembrane domain predicted. Clusters 4 and 13 showed the expected expression pattern for those genes that could encode heme export proteins, with 197 (93+104 respectively) genes identified with 1+ predicted TM domains. In addition to observing expression changes due to heme concentration, changes in nutrient content can also be examined as fetal bovine serum was only present in the first set of samples (Clusters 1, 2, 6, 7 & 11). When the DEGs were compared back to those genes found in the import-like clusters, only 25.2% of the genes upregulated during heme deficiency conditions and 32.6% of the genes downregulated during heme overexposure conditions were also found in an import-like cluster (Fig. S10A). Like with the clusters showing the expected expression pattern of heme import genes, very few genes were shared between the clusters showing the heme export expression pattern and those in the DE analysis, with only 19.7% shared of those downregulated during heme deficiency and 14.8% shared of those upregulated during heme overexposure (Fig. S10B). Of the 13 genes identified as genes encoding for potential heme membrane bound transporters, only 1 was not also found in the cluster analysis, AAEL000242; 6 of the 7 genes with expression patterns consistent with heme export were found in cluster 4 (5 genes) and 13 (1 gene) and all of the 6 genes with expression patterns consistent with heme import were found in the import-like clusters 5 (2) & 8 (4). This list can be further reduced by examination of the predicted functions of these genes, finding only 10 genes (5 &5 respectively) with either unknown or transport related function.

**References**

1. Xiao G, Wan Z, Fan Q, Tang X, Zhou B. **The metal transporter ZIP13 supplies iron into the secretory pathway in *Drosophila melanogaster***. eLife. 2014;3.

2. Tsujimoto H, Anderson MAE, Myles KM, Adelman ZN. **Identification of Candidate Iron Transporters From the ZIP/ZnT Gene Families in the Mosquito *Aedes aegypti***. Front Physiol. 2018;9:380.
