# Supplementary material for: Transcriptomic analyses of Aedes aegypti cultured cells and ex vivo midguts in response to an excess or deficiency of heme: a quest for transcriptionally-regulated heme transporters
Source: BMC Genomics. 2020 Aug 31;21:604. doi: 10.1186/s12864-020-06981-5 (PMC7460771; doi:10.1186/s12864-020-06981-5)
Supplement: Supplementary file 4 — Additional file 4 Fig. S1. Treatment conditions of A20 and Aag2 mosquito cells prior to RNAseq analysis. Schematic representation of the various treatments used to prepare samples for RNAseq. Cell type (Aag2/A20), incubation time (48 h, 72 h), growth media type (L-15, Schneider's Drosophila), and heme supplement (0 μM, 10 μM, 20 μM), with (Normal media, indicated by 50 mL conical tube) or without (indicated by mini centrifuge tube) FBS present in the media. Schematic was generated using Biorender through a license from Texas A&M University. Fig. S2. Multidimensional Scaling Plot of RNAseq data derived from Aag2 cultured cells grown in Schneider’s Drosophila medium. Multidimensional scaling plot displaying transcriptomic changes in Aag2 cells grown in Schneider’s Drosophila medium exposed to heme overload or heme deficiency conditions. The cells grown in normal growth media are circled in blue (FBS), the cells exposed to heme overload are circled in green (10 μM Heme) and the cells exposed to heme deficiency are circled in orange (0 μM Heme). Fig. S3. RNAseq-based transcriptomic analyses after 48-h heme treatment in Aag2 cells. (A) Multidimensional scaling plot. The FBS treated group is circled in blue and the FBS + 20 μM heme group is circled in green. (B) Log2 fold change (logFC) vs Log10 counts per million (logCPM) plots of expressed genes; genes with an adjusted p-value corrected for multiple testing (False discovery rate: FDR) of < 0.0001 are shown in red. (C) Volcano plot of genes expressed in analysis. (D) 6 distinct gene expression profile clusters were identified through soft clustering. Only those genes with membership scores > 50% are shown in each cluster. Red lines show high membership in a cluster while blue indicates low membership. Heme import-like clusters are highlighted in red and export-like clusters in blue. The different heme treatment groups are given on the X-axis of each cluster; n, the number of genes present in a cluster. Fig. S4. Multidime [file 12864_2020_6981_MOESM4_ESM.pptx]

## Slide 1
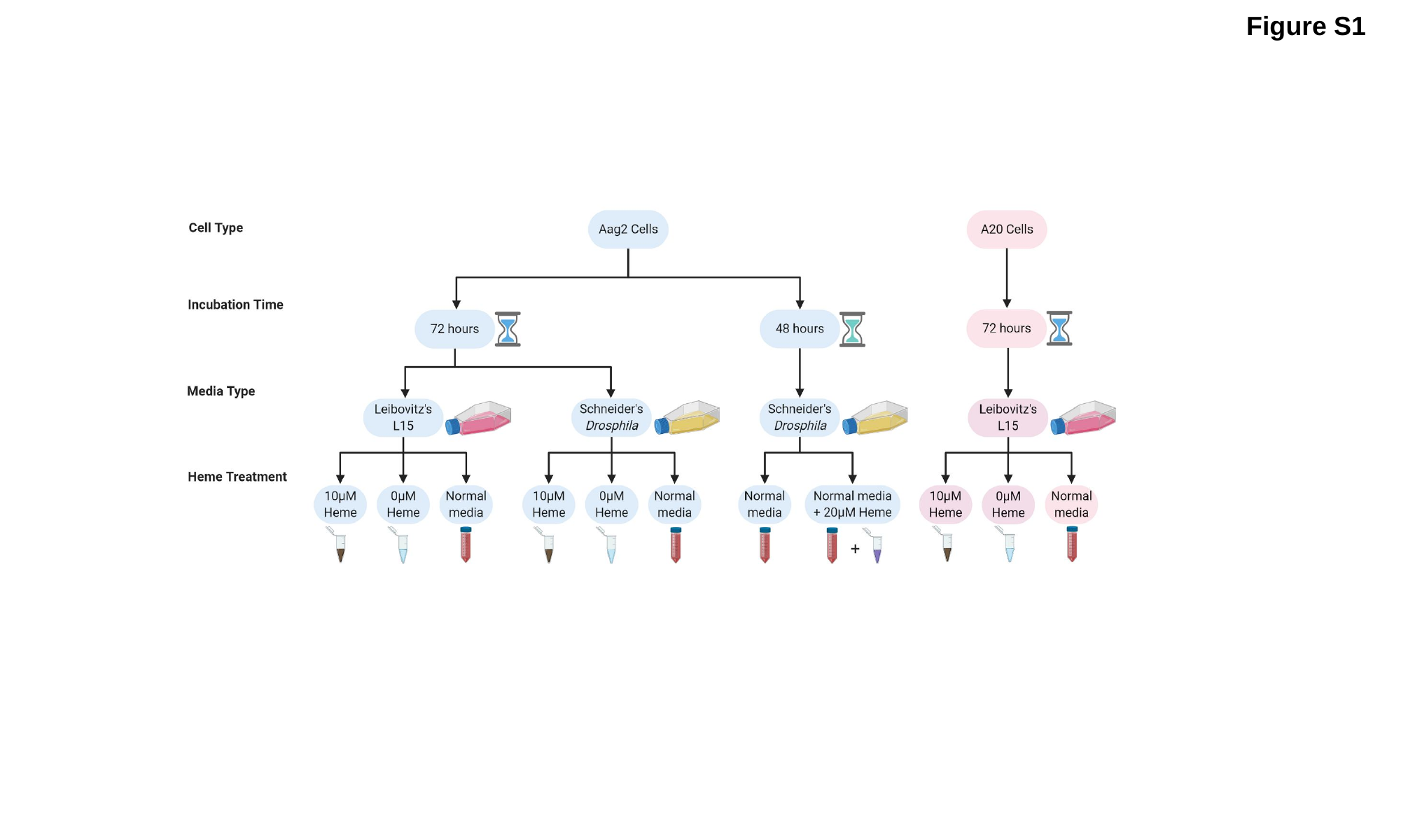

Figure S1

## Slide 2
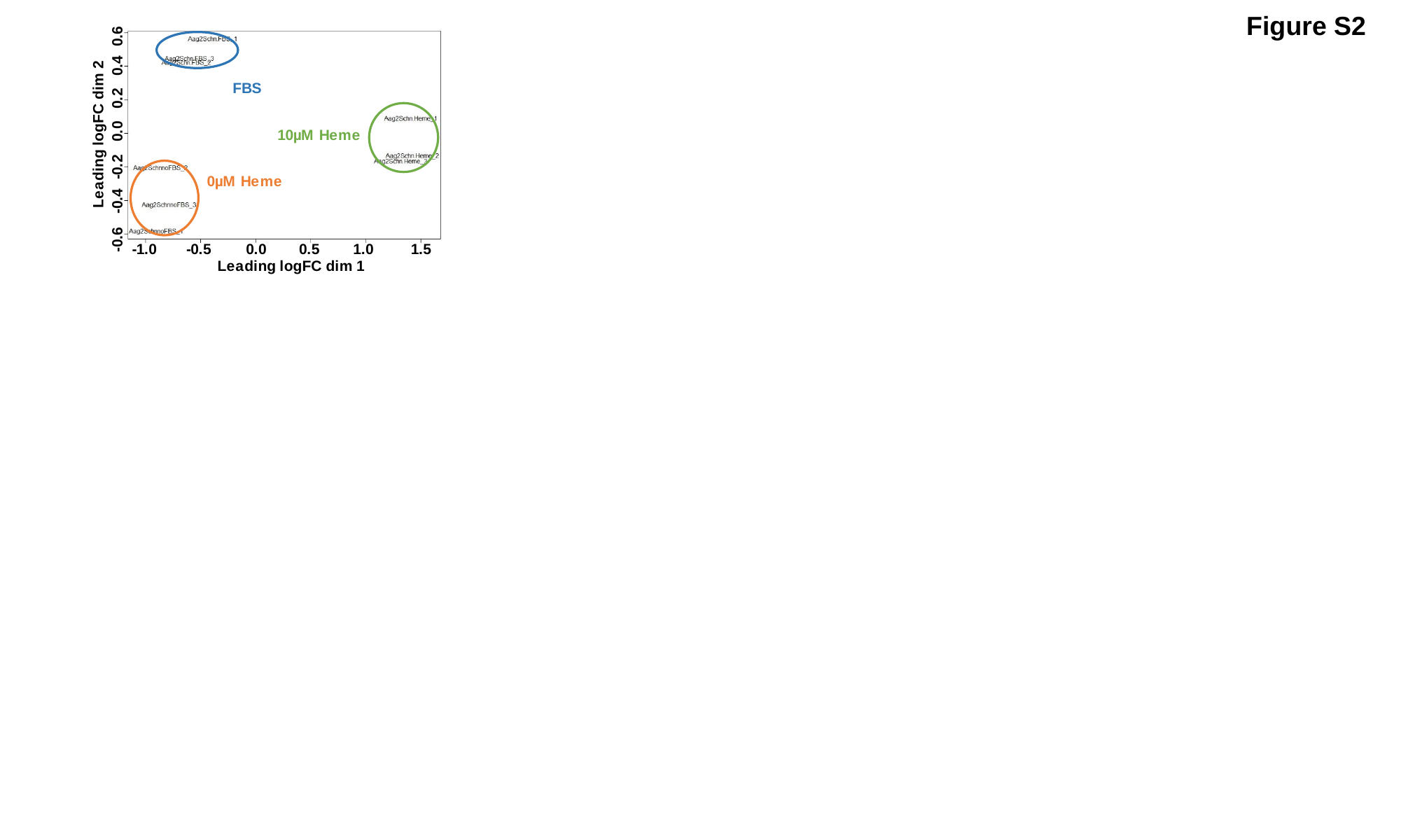

Figure S2

## Slide 3
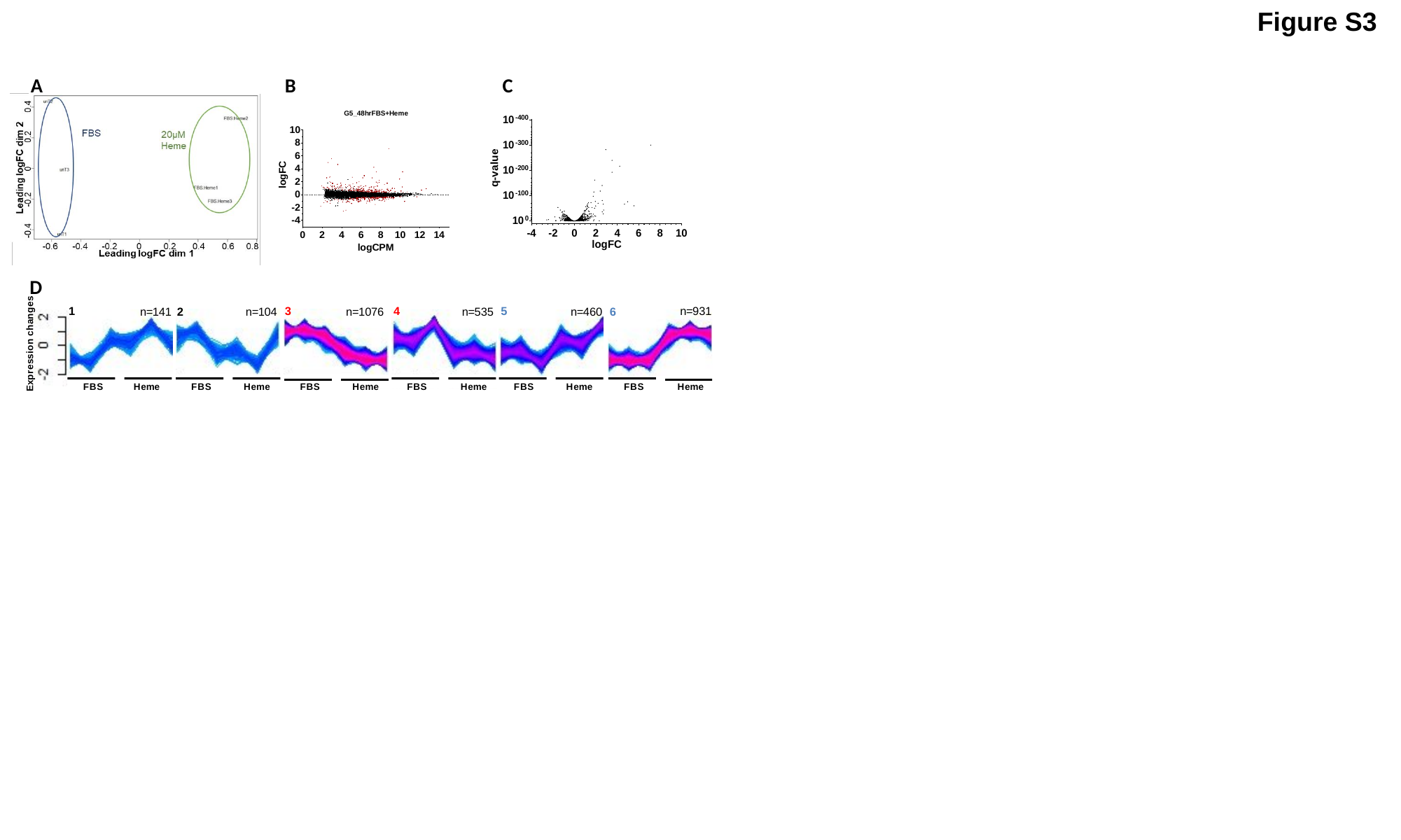

Figure S3

## Slide 4
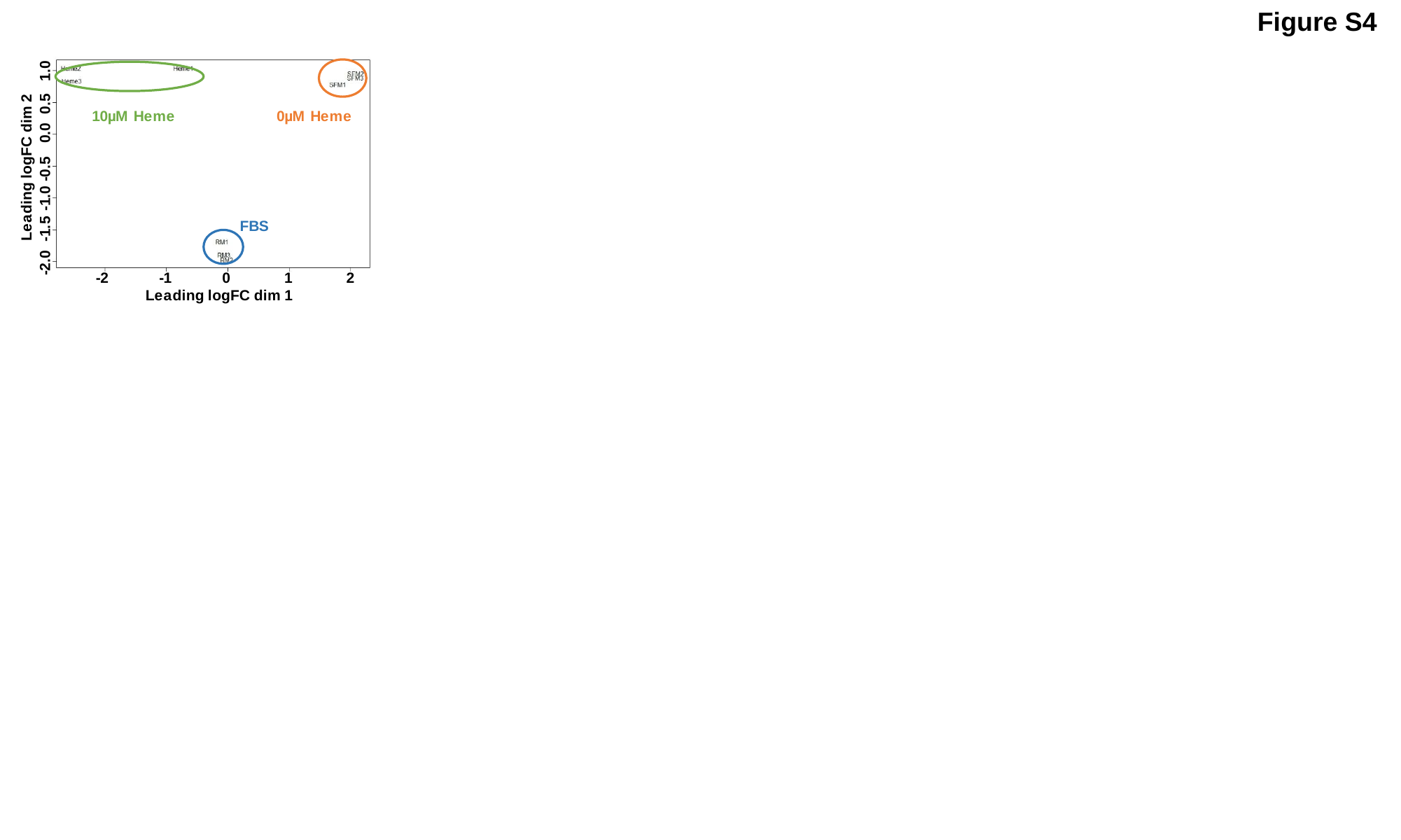

Figure S4

## Slide 5
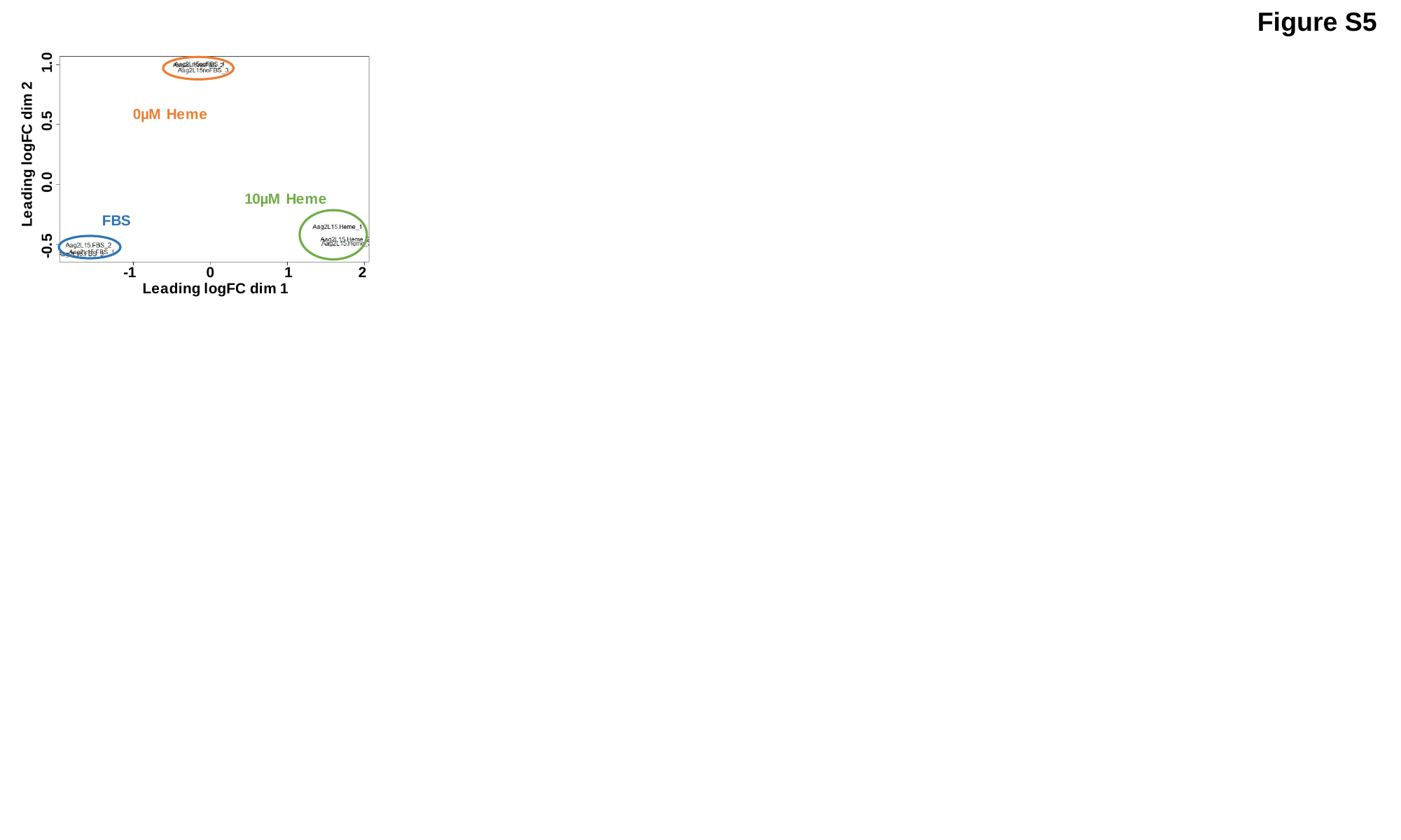

Figure S5

## Slide 6
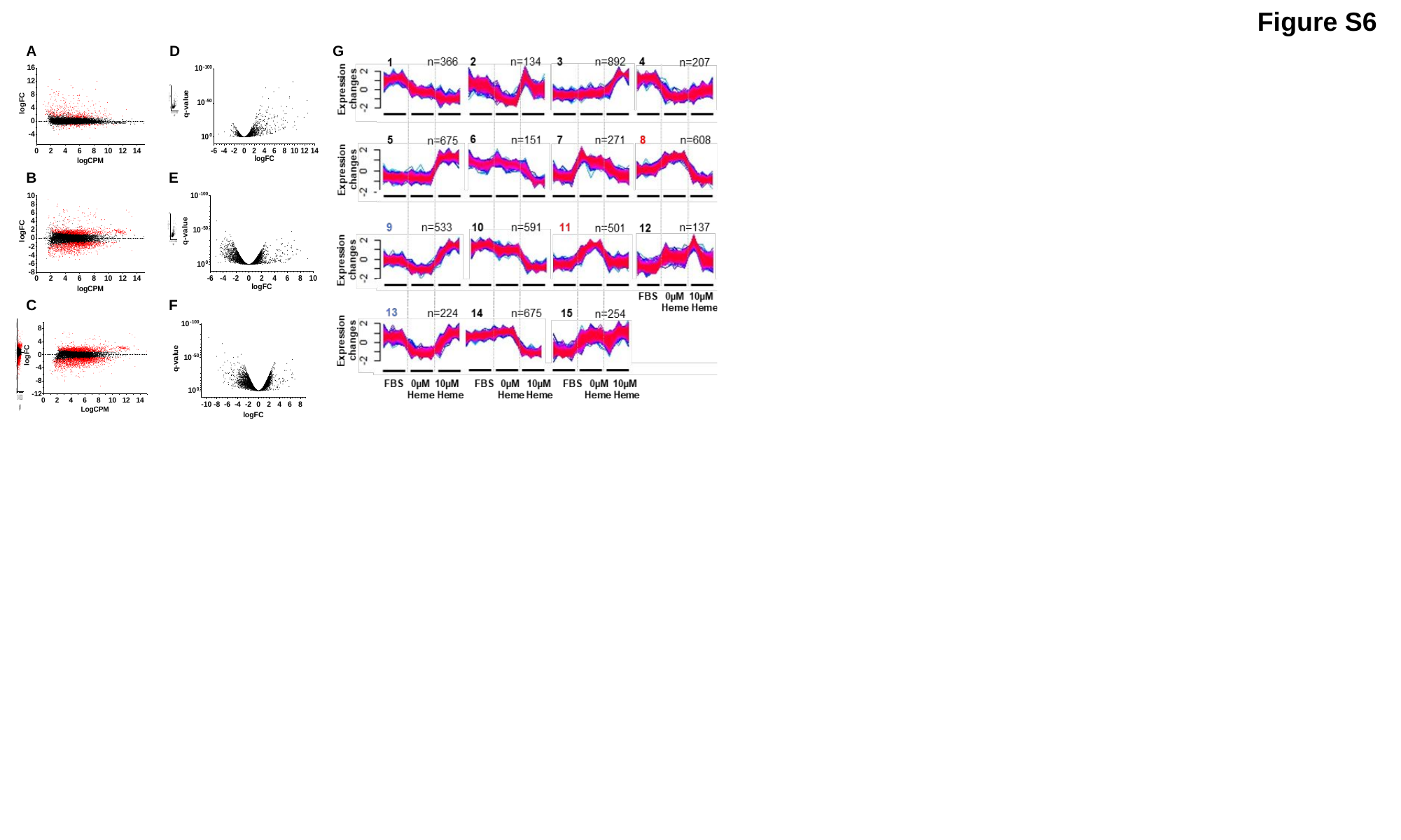

Figure S6

## Slide 7
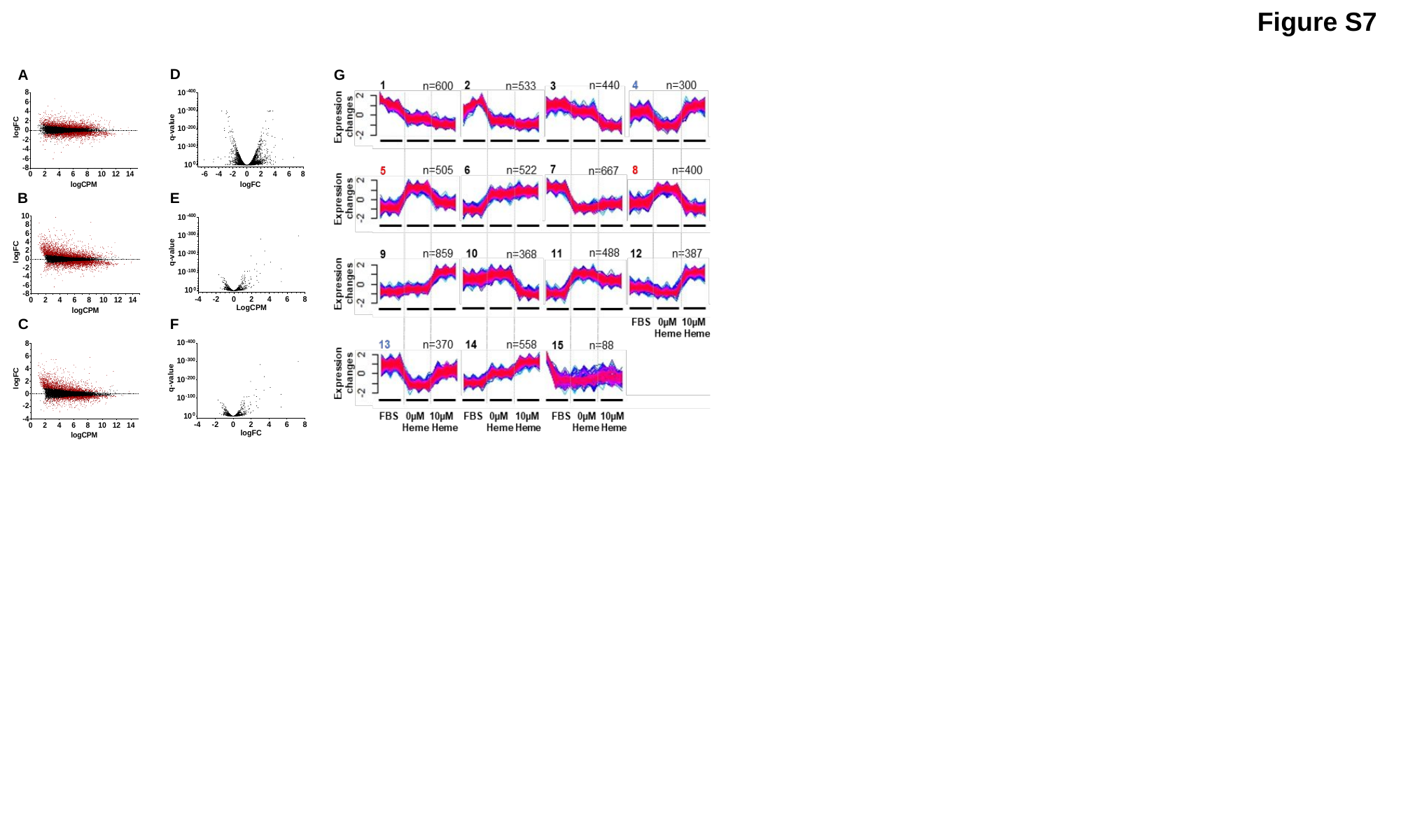

Figure S7

## Slide 8
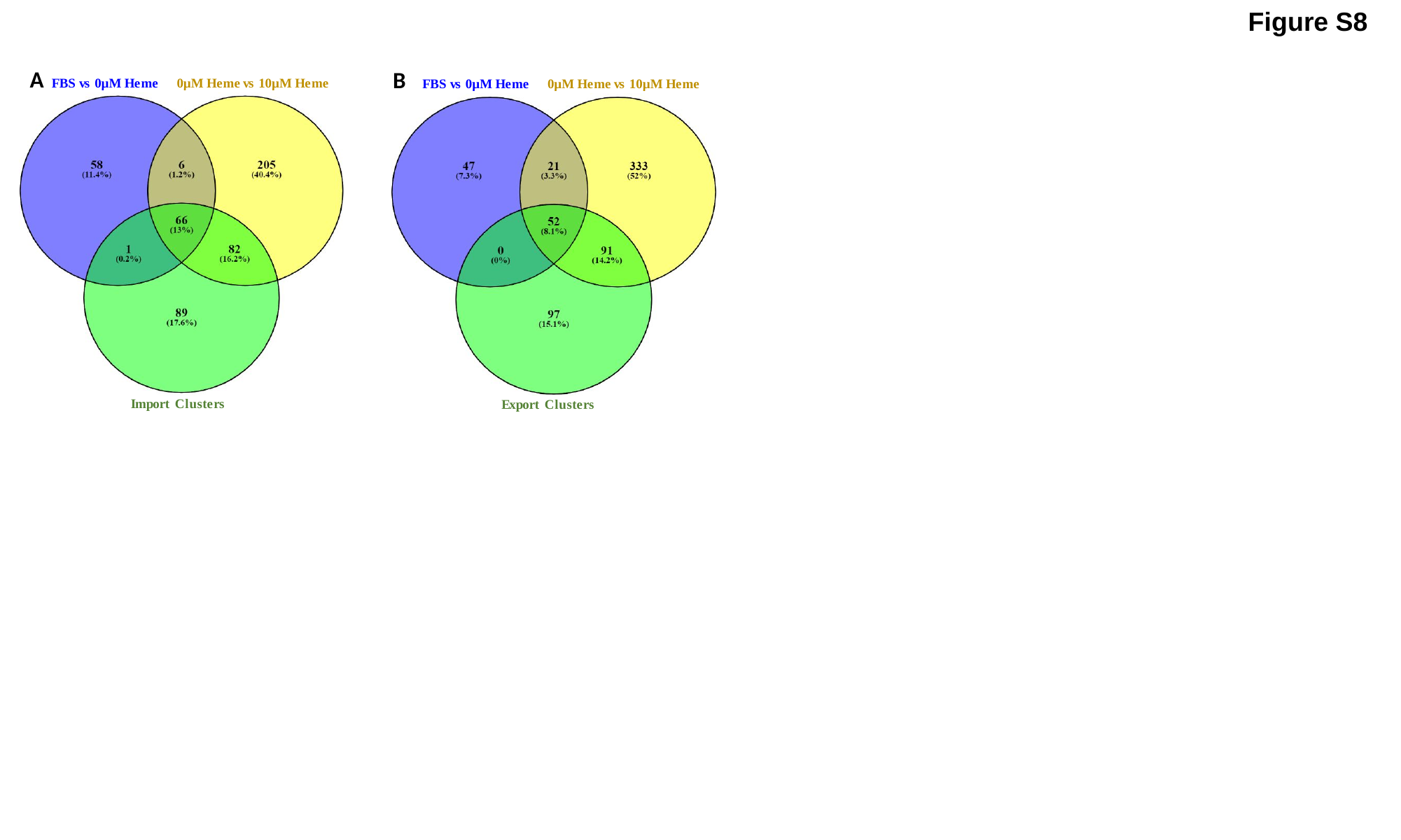

Figure S8

## Slide 9
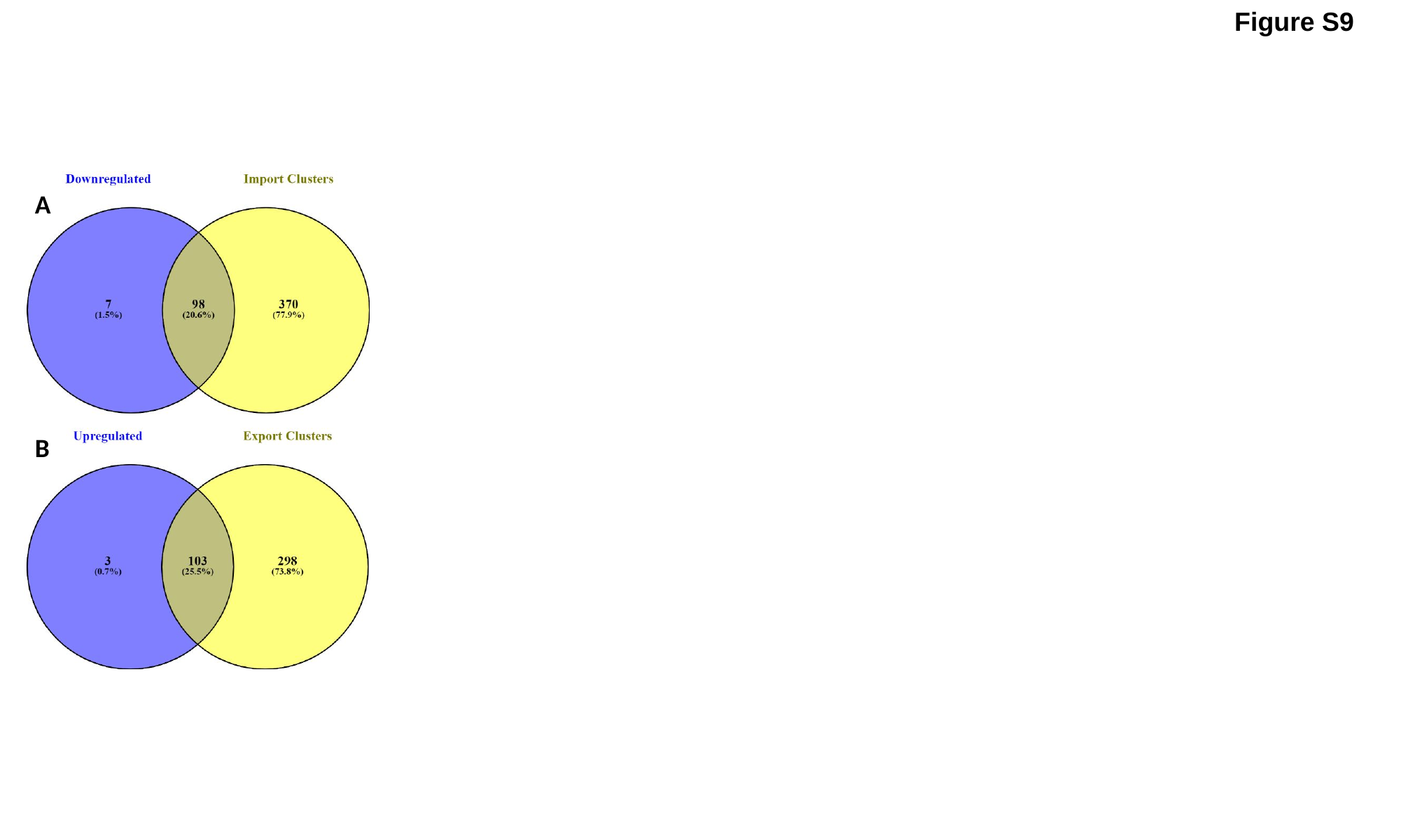

Figure S9

## Slide 10
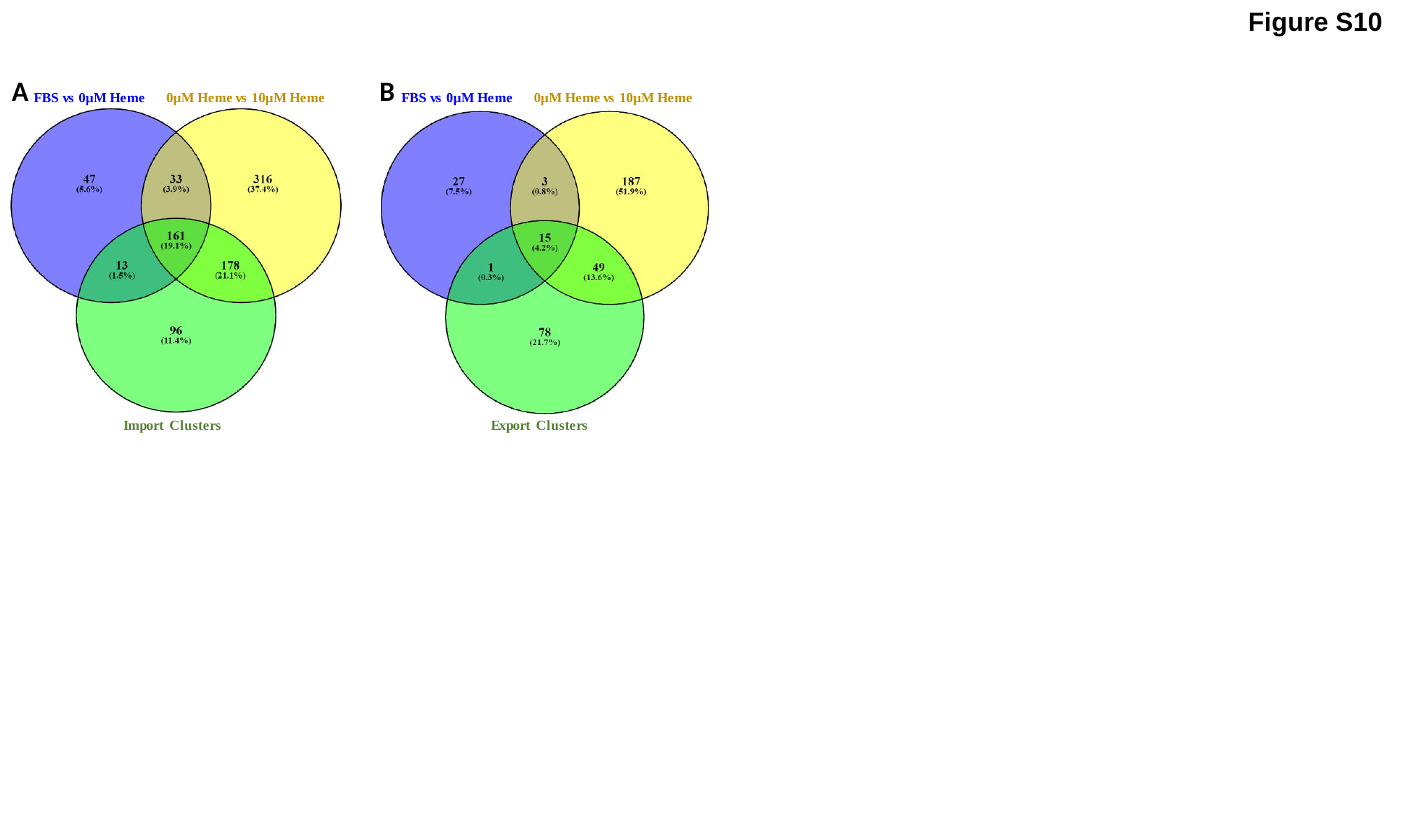

Figure S10

## Slide 11
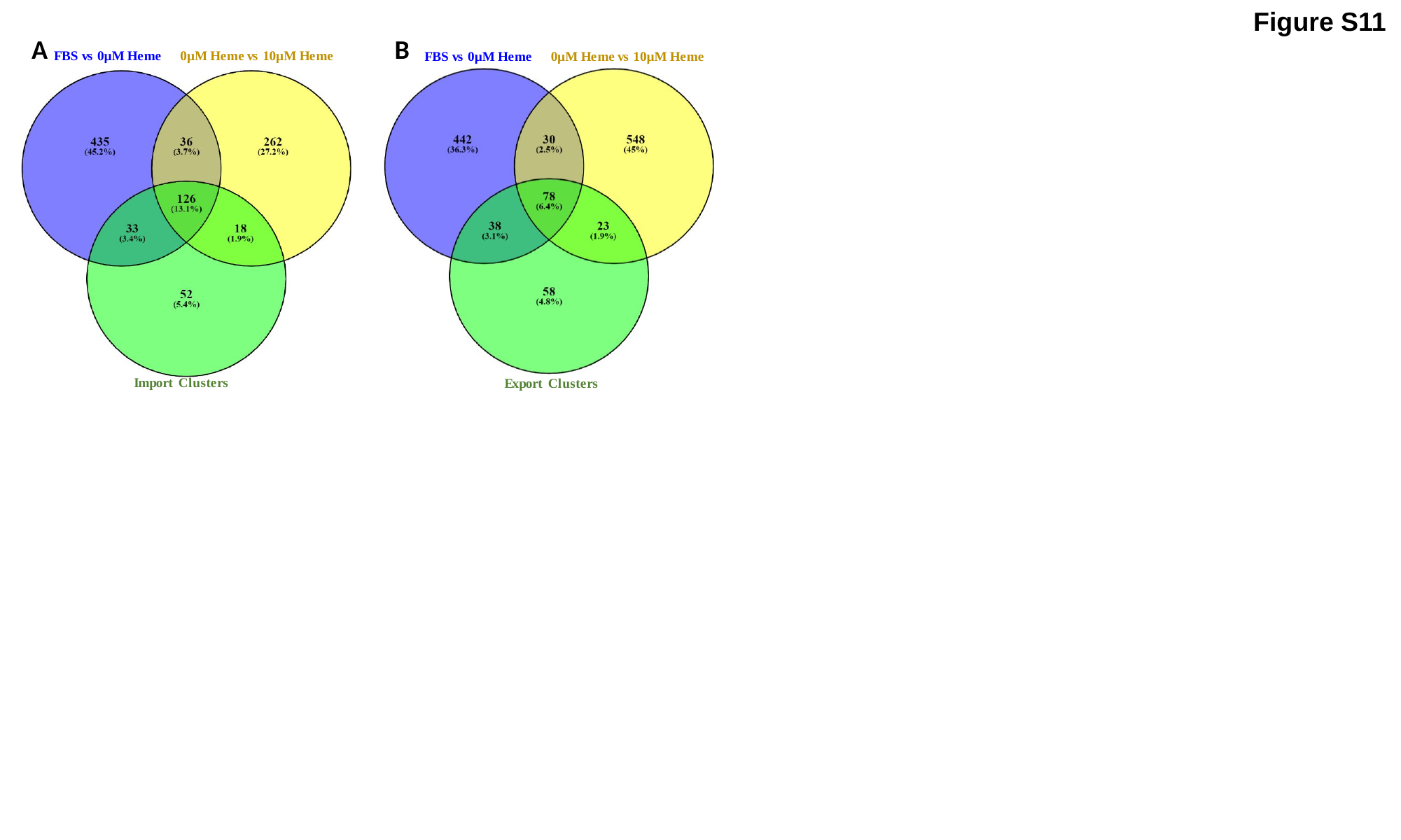

Figure S11

## Slide 12
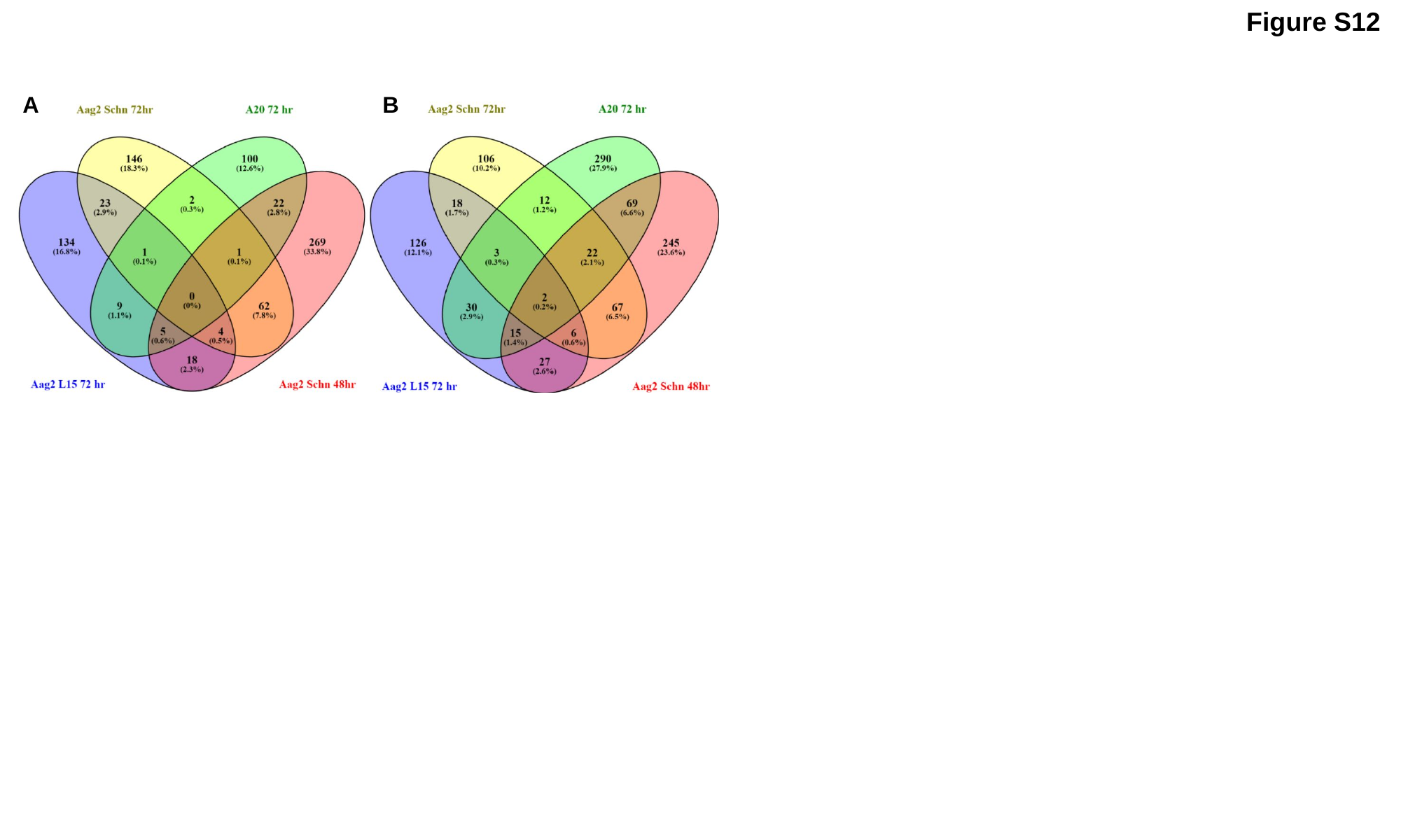

Figure S12

## Slide 13
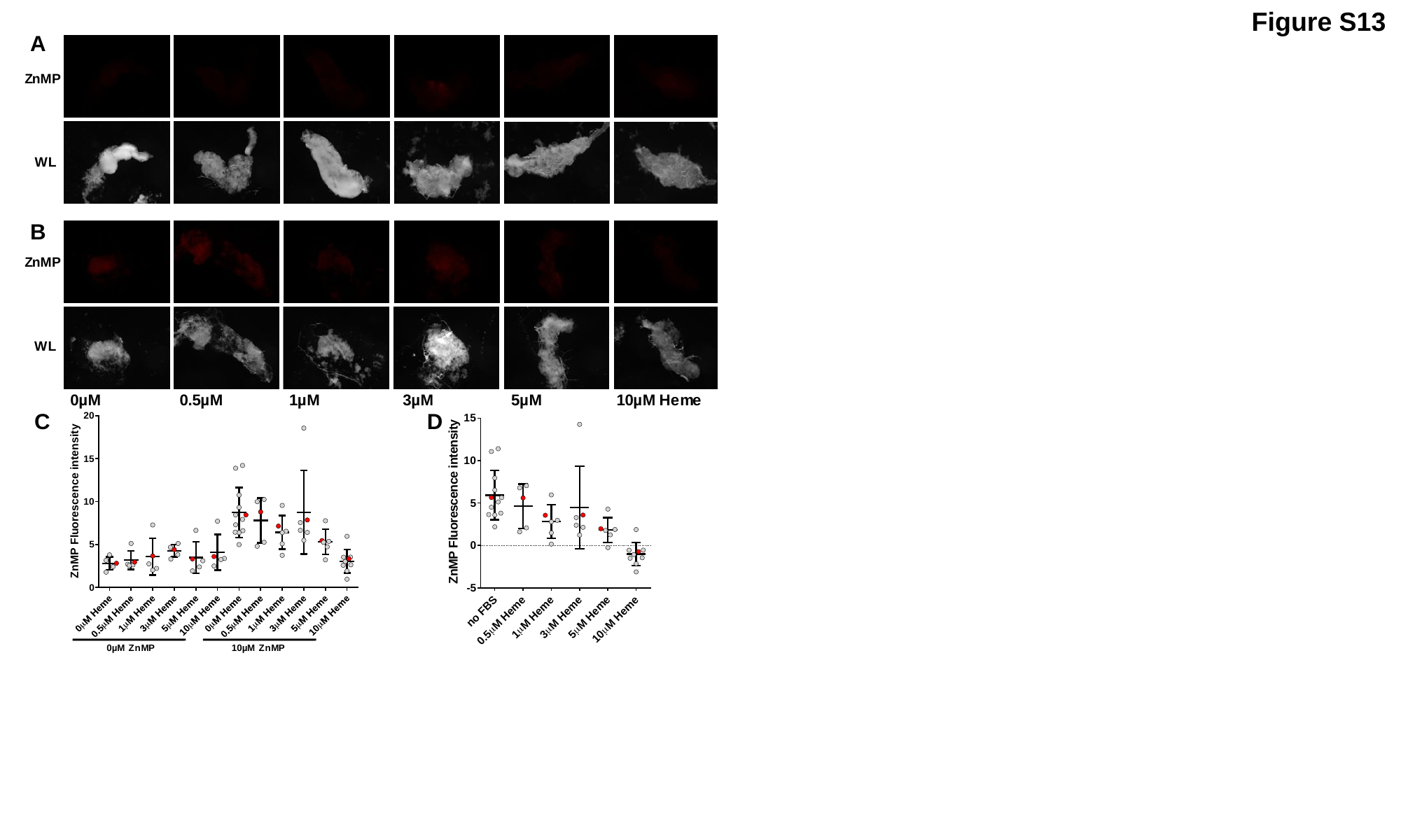

Figure S13

## Slide 14
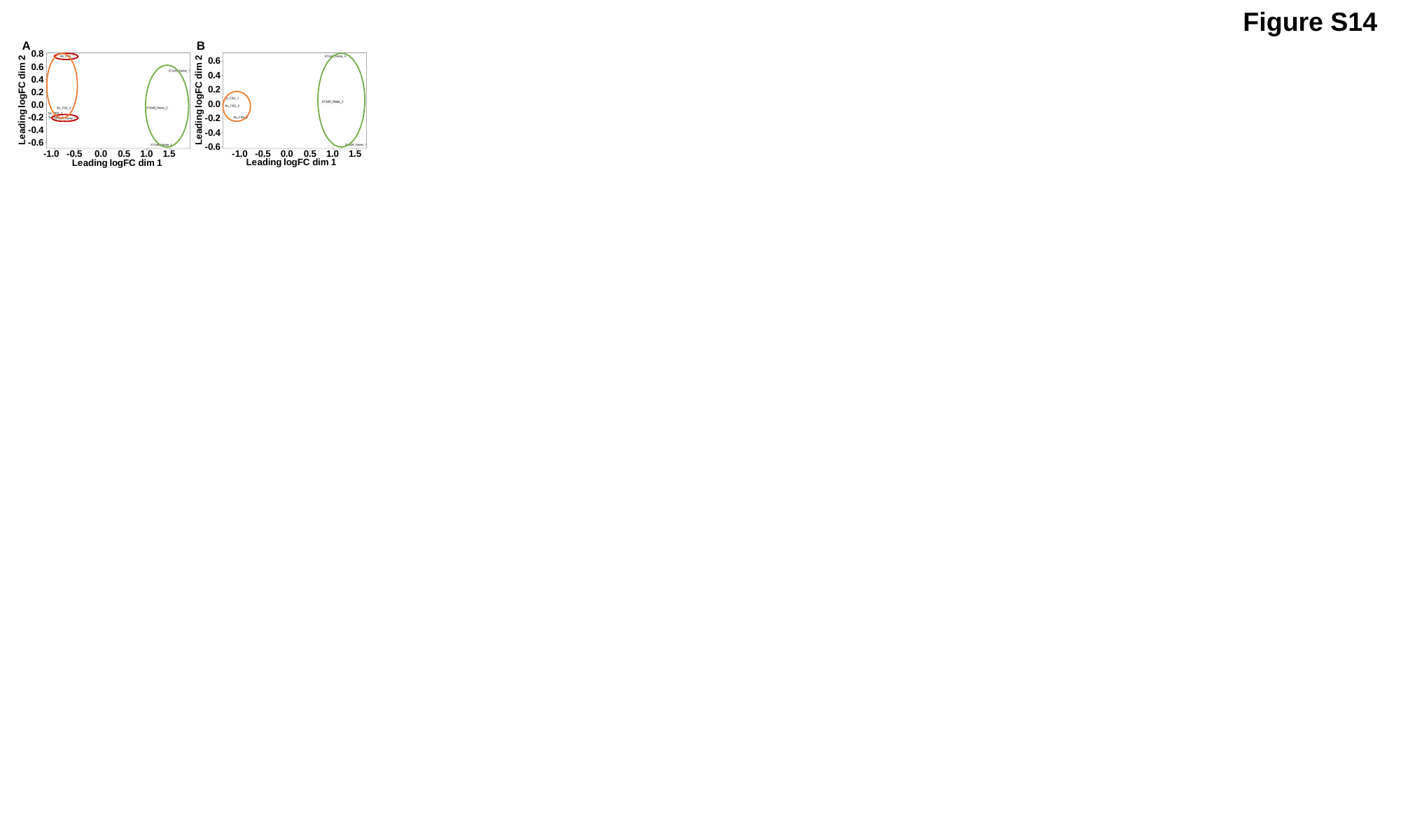

Figure S14
